# Lirilumab and Avelumab Enhance Anti-HPV+ Cervical Cancer Activity of Natural Killer Cells *via* Vav1-Dependent NF-κB Disinhibition

**DOI:** 10.3389/fonc.2022.747482

**Published:** 2022-01-31

**Authors:** Hongli Liu, Sihui Zhou, Jing Liu, Fuliang Chen, Yuan Zhang, Mengjun Liu, Shengping Min, Hongtao Wang, Xiaojing Wang, Nan Wu

**Affiliations:** ^1^ Department of Gynecological Oncology, First Affiliated Hospital of Bengbu Medical College, Bengbu, China; ^2^ Anhui Clinical and Preclinical Key Laboratory of Respiratory Disease, Molecular Diagnosis Center, Department of Pulmonary and Critical Care Medicine, First Affiliated Hospital of Bengbu Medical College, Bengbu, China; ^3^ Anhui Province Key Laboratory of Immunology in Chronic Diseases, Department of Laboratory Medicine, Bengbu Medical College, Bengbu, China

**Keywords:** cervical cancer, HPV, immunotherapy, NK cell, NF-κB

## Abstract

**Background:**

We investigated the efficacy and mechanism of the anti-KIR immunotherapy lirilumab and anti-PD-L1 immunotherapy avelumab on natural killer (NK) cell activity against HPV+ cervical cancer.

**Methods:**

NK cell-mediated lysis of autologous biopsy-derived malignant cervical squamous cells and normal cervical squamous cells were measured by europium-release cytotoxicity assays. Cytokine and granzyme B release were measured by ELISPOT effector-cell-based assays and ELISA. Murine cervical cancer tumor models were constructed to assess implanted tumor volumes over time and intratumoral immune cell infiltration. Receptor-crosslinking and plate-immobilized antibody stimulation studies, with or without p65 and Vav1 silencing, were used to investigate NF-κB pathway disinhibition in NK cells.

**Results:**

Lirilumab and avelumab each enhanced NK cell disinhibition and NK cell-mediated lysis of autologous cervical cancer cells *in vitro* while reducing HPV+ tumor volumes and increasing intratumoral NK cell infiltration and cytolysis *in vivo*. Moreover, lirilumab and avelumab each promoted NK cell NF-κB disinhibition as well as stimulated cytokine and granzyme B expression in a NF-κB-dependent manner. Lirilumab+avelumab enhanced all aforementioned effects compared to either monotherapy. Vav1 silencing eliminated disinhibition of NF-κB signaling by lirilumab and avelumab, indicating their disinhibiting effects are Vav1-dependent.

**Conclusions:**

This study supports a novel approach to enhancing NK cell lysis against HPV+ cervical cancer cells through combining lirilumab and avelumab.

## Introduction

Cervical cancer is the fourth-highest ranking malignancy in terms of incidence among women globally (1), and human papillomavirus (HPV) is the predominant cause of cervical cancer (2). Due to disease relapse and late-stage diagnosis, the prognosis for this malignancy remains extremely poor, resulting in this disease being the leading cause of mortality for gynecologic cancers worldwide (1). Therefore, the development of more effective therapeutics for HPV+ cervical cancer remains a pressing clinical need.

Natural killer (NK) cells, when activated by surface receptor cues, play a key role against malignant tumors by directly killing transformed cells with proteolytic granzymes and secreting immunoregulatory cytokines, such as interferon (IFN)-γ, macrophage inflammatory proteins (MIPs), interleukins (IL-8, IL-10), and TNF-α (3, 4). Release of these cytokines recruits other immune cells (e.g., T-helper 1 [Th1] cells, myeloid cells) to the site and induces their anti-tumor response (3, 4). Notably, upregulation of the NK cell’s programmed cell death protein-1 receptor (PD-1) and tumor cell’s PD-1 ligand 1 (PD-L1, B7-H1) have been associated with HPV+ cervical cancer cases, with higher PD-1 and PD-L1 correlating with heightened cervical intraepithelial neoplasia (CIN) grade (5–7). This evidence suggests that dysregulated PD-1/PD-L1-mediated immunity may contribute to HPV-associated cervical oncogenesis, making anti-PD-1/PD-L1 immunotherapies particularly promising for HPV+ cervical cancer patients. Specifically, monoclonal antibodies (mAbs) have been developed to enhance the innate NK cell response against cervical cancer cells, including the anti-PD-1 mAbs nivolumab and pembrolizumab and the anti-PD-L1/2 mAbs MPDL3280A and AMP-224 (8, 9). These antibodies inhibit the interaction between PD-L1 on tumor cells and its receptor PD-1 on NK cells, thereby abrogating the immunosuppressive signal and enhancing immune cell disinhibition (10, 11). One such mAb – MSB0010718C (termed avelumab) – is a human IgG1-based anti-PD-L1 mAb that has shown positive benefits across several tumor types (12), and therefore, may also show promise in HPV+ cervical cancer patients.

In addition, the killer cell immunoglobulin-like receptor (KIR) family of NK cell receptors and their human leukocyte antigen C (HLA-C) ligands also interact to suppress NK cell function. As HPV+ invasive cervical cancer patients display upregulation in HLA-C/KIR2DL2 and HLA-C/KIR2DL3 antigen pairs (13), enhancing the HLA-C/KIR interaction may suppress normal NK cell function in HPV+ cervical cancer patients. The human anti-KIR mAb lirilumab (IPH2102, BMS-986015) has been shown to block HLA-C from binding to KIR2DL1, -L2, and -L3 receptors, thereby impairing their inhibitory signaling on NK cells (14). Indeed, anti-KIR mAb immunotherapy results in upregulation of several key cytokines (15, 16) and has shown positive benefits across several tumor types (14, 17). Therefore, we hypothesized that targeting the HLA-C/KIR interaction with lirilumab may represent another novel immunotherapeutic strategy for HPV+ cervical cancer.

Here, we provide novel evidence that lirilumab’s KIR blockade and avelumab’s PD-L1 blockade individually promote NK cell activity against HPV+ cervical cancer cells while sparing normal cells. Moreover, combining lirilumab with avelumab promoted NK cell activity against HPV+ cervical cancer cells, resulting in HPV+ tumor regression and enhanced intratumoral NK cell infiltration and cytolysis. As NF-κB signaling has been shown to play a critical role in receptor-regulated NK cell activity (18), we also discovered the cooperative disinhibition of NF-κB signaling by lirilumab and avelumab in NK cells, converging at the level of Vav1 and the downstream NF-κB p65 subunit.

## Methods

All procedures involving patient sample collection and *in vivo* animal experimentation were approved by the Ethics Review Committee of the First Affiliated Hospital of Bengbu Medical College (approval no. 2020-088). All human subjects recruited for this study provided written informed consent prior to participation. The animal procedures were in accordance with the standards set forth in the NIH Guide for the Care and Use of Laboratory Animals (Bethesda, MD).

The detailed procedures regarding patient sample collection, monoclonal antibodies, NK cell isolation, antibody binding and antigen expression assays, cell mixing, europium-release cytotoxicity assays, ELISPOT assays, NK cell CD107a expression by flow cytometry, construction of HPV+ murine cervical cancer cell line, syngeneic murine tumor model, scoring of immune cell infiltration within murine tumors, IL-2 priming of NK cells, receptor-crosslinking and plate-immobilized antibody stimulation, fractionation and immunoblotting, NF-κB DNA binding assays, NF-κB reporter assays, supernatant ELISA, RNA silencing, and quantitative real-time RT-PCR are provided in the **Supplementary Methods**.

All data are reported as means and associated standard deviations (SDs) derived from at least three independent experiments. Comparisons between two experimental conditions were analyzed using Student’s *t-*test, while comparisons among three or more experimental conditions were analyzed by one-way ANOVA with a Bonferroni *post-hoc* correction. The statistical significance threshold for all comparisons was set at 0.05.

## Results

### Lirilumab and Avelumab Promote NK Cell Disinhibition and Cytotoxicity Against Human Cervical Cancer Cells

**Figure 1:**
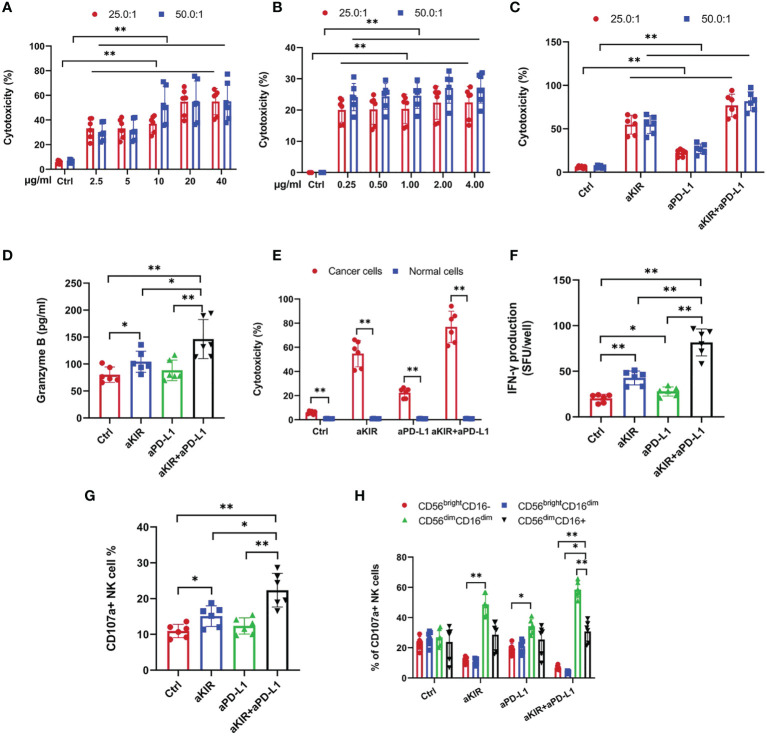
Lirilumab and Avelumab Promote NK Cell Disinhibition and Cytotoxicity against HPV+ Cervical Cancer Cells *in vitro*. Blood-derived NK cells and autologous biopsy-derived malignant squamous cells obtained from HPV+ cervical cancer patients (n = 24) were incubated with DMSO (control), lirilumab (aKIR, 20 µg/ml) and/or avelumab (aPD-L1, 2.0 µg/ml), respectively, for 24 hours and then cultured together for 30 min. **(A-C)** NK cell-mediated lysis of cancer cells as measured by a target-based, europium-release cytotoxicity assay at E:T ratios of 25:1 and 50:1. **(D)** Blood-derived NK cells and autologous biopsy-derived malignant squamous cells or normal cervical squamous cells were incubated with control, aKIR, and/or aPD-L1 for 24 hours and then cultured together for 30 min for NK cell-mediated cytotoxicity assays. **(E, F)** NK cell **(E)** granzyme B and **(F)** IFN-γ production as measured by the ELISPOT effector-cell-based assays. **(G)** Percentages of CD107a+ NK cells. **(H)** Percentages of CD56^dim^CD16^dim^ and CD56^dim^CD16+ CD107a+ NK cells. *P < 0.05, **P < 0.01 [**(A–C)** two-way ANOVA, **(A, B)** mAb concentration factor × E:T ratio factor, **(C)** mAb factor × E:T ratio factor, **(D, F–H)** one-way ANOVA, or **(E)** Student’s *t*-test]. n=3 biological replicates×3 technical replicates.

We investigated the effects of lirilumab (aKIR) and avelumab (aPD-L1) upon NK cell-mediated lysis of autologous malignant squamous cells obtained from HPV+ cervical cancer patients using a europium-release cytotoxicity assay at E:T ratios of 25:1 and 50:1. Lirilumab or avelumab each promoted NK cell-mediated lysis of cancer cells in a dose-dependent manner (**Figures 1A, B**). Moreover, lirilumab+avelumab promoted NK cell-mediated lysis of cancer cells compared to either monotherapy (**Figure 1C**). Additional NK cell-mediated lysis experiments across three additional co-culturing timepoints (60 min, 120 min, and 180 min) revealed similar results (**Supplementary Figure S2**).

Notably, the aforementioned NK cell-mediated lytic effects were not observed with normal cervical squamous cells (**Figure 1D**). Additional NK cell-mediated lysis experiments for three additional co-culturing timepoints (60 min, 120 min, and 180 min) at both 25:1 and 50:1 E:T ratios revealed similar results (**Supplementary Figure S3**). As NK cells have been shown to upregulate surface PD-L1 expression following exposure to myeloid leukemia cells (19), we hypothesized that the aforementioned differential effect may be due to the presence of PD-L1 expression on NK cells exposed to cervical cancer cells but absent or *de minimus* PD-L1 antigen expression on NK cells exposed to normal cervical cells. However, flow cytometry revealed negligible surface PD-L1 expression on NK cells following exposure to either cancerous or benign cervical cells (**Supplementary Figure S4**). Alternatively, we hypothesized that this differential effect may be due to the presence of PD-L1 expression on cervical cancer cells but absent or *de minimus* PD-L1 antigen expression on normal cervical cells. Indeed, flow cytometry revealed positive surface PD-L1 expression on cervical cancer cells but negligible surface PD-L1 expression on normal cervical cells (**Supplementary Figure S5**).

We investigated the effects of lirilumab+avelumab upon NK cell disinhibition using NK cells mixed with autologous malignant squamous cells obtained from HPV+ cervical cancer patients. Lirilumab+avelumab enhanced NK cell granzyme B and IFN-γ production compared to either monotherapy (**Figures 1E, F**). As surface expression of the lytic granule protein CD107a is a reliable marker of NK cell degranulation activity (20), lirilumab+avelumab raised the percentages of CD107a+ NK cells and more mature, cytolytic CD107a+ NK cell subsets (i.e., CD56^dim^CD16^dim^) (21) compared to either monotherapy (**Figures 1G, H**).

### Lirilumab Analog and Avelumab Analogue Enhance Anti-Tumor Activity *In Vivo*

We investigated the anti-tumor effects of the murine lirilumab analogue aLy49 and the murine avelumab analogue aPdl1 upon several syngeneic murine HPV+ cervical tumor model (**Supplementary Figure S6**). The U14 cell line was selected as it is syngeneic with the Kunming mice hosts (22); the U14 cells were transfected with the HPV16 E6 and E7 genes to create a murine HPV+ cervical cancer cell line (23). The tumor sizes at the time of therapy initiation for all models are provided in **Supplementary Table S1**.

**Figure 2:**
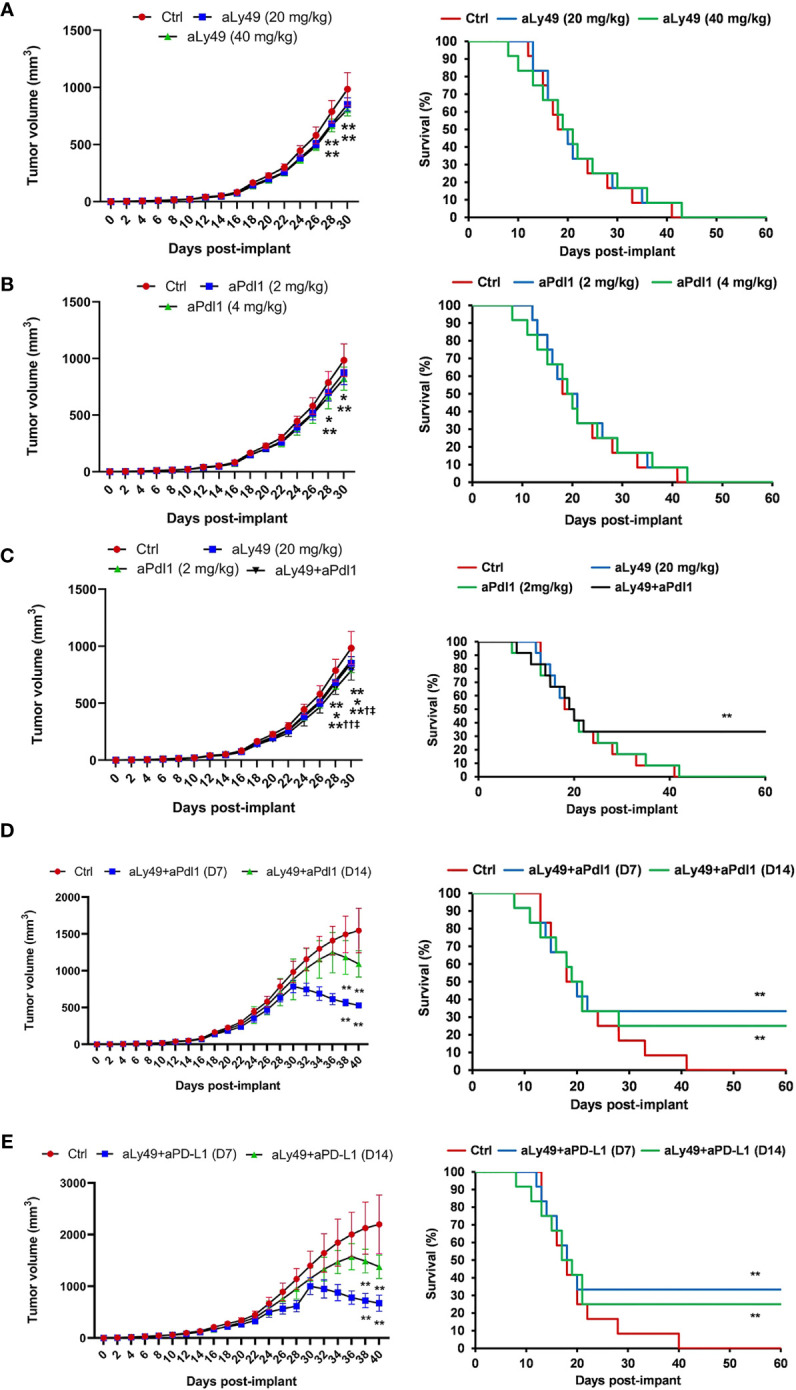
Lirilumab and Avelumab Enhance Anti-Tumor Activity *in vivo*. **(A)** Tumor-bearing mice administered IgG (control), aLy49 (20 mg/kg daily), or aLy49 (40 mg/kg daily) intraperitoneally (i.p.) seven days (D7) after HPV+ U14 cervical tumor cell implantation. **(B)** Tumor-bearing mice administered IgG (control), aPdl1 (2 mg/kg daily), or aPdl1 (4 mg/kg daily) i.p. seven days after HPV+ U14 cell implantation. **(C)** aLy49 (20 mg/kg daily) plus aPdl1 (2 mg/kg daily) starting on D7. **(D)** Tumor-bearing mice administered IgG (control) or aLy49 (20 mg/kg daily) plus aPdl1 (2 mg/kg daily) i.p. starting at D14. **(E)** Tumor-bearing mice were administered IgG (control) or aLy49 (20 mg/kg daily) plus aPdl1 (2 mg/kg daily) i.p. starting at D7 or D14. *P < 0.05, **P < 0.01 versus control group, ^†^P < 0.05, ^††^P < 0.01 versus aLy49 group, ^‡^P < 0.05 versus aPdl1 group [(left) one-way ANOVA or (right) log-rank test]. *n=*12 mice per cohort.

A set of tumor-bearing mice were administered aLy49 (20 or 40 mg/kg daily) intraperitoneally (i.p.) starting on day 7 (D7) after HPV+ U14 cervical tumor cell implantation (**Supplementary Figure S6A**). aLy49 reduced tumor volume at day 30 but had no impact on survival (**Figure 2A**). aPdl1 (2 or 4 mg/kg daily) i.p. starting on D7 (**Supplementary Figure S6B**). aPdl1 reduced tumor volume at day 30 but had no impact on survival (**Figure 2B**). aLy49 (20 mg/kg daily) plus aPdl1 (2 mg/kg daily) simultaneously administered starting on D7 (**Supplementary Figure S6C**) reduced tumor volume at day 30 compared to either monotherapy and significantly increased the 60-day pos*t-*implant survival (**Figure 2C**). aLy49-treated and aPdl1-treated tumors showed higher levels of T-cell infiltration, overall immune cell infiltration, CD8+ T-cell infiltration, CD4+ Treg infiltration, CD4+ effector T-cell infiltration, and CD107a+ NK cell infiltration (**Supplementary Figures S7A–F**). Consistently, aLy49-treated and aPdl1-treated tumors displayed enhanced granzyme B expression and cytolytic scores (**Supplementary Figures S7G–H**). aLy49+aPdl1 enhanced T-cell infiltration, CD8+ T-cell infiltration, CD4+ effector T-cell infiltration, CD107a+ NK cell infiltration, and cytolysis (**Supplementary Figure S7**).

A set of tumor-bearing mice were simultaneously administered aLy49 (20 mg/kg daily) plus aPdl1 (2 mg/kg daily) i.p. starting at D14 to test anti-tumor activity on larger tumors (**Supplementary Figure S6D**). aLy49+aPdl1 administered starting on D14 enhanced reductions in tumor volume at day 30 and significantly increased 60-day post-implant survival rates (**Figure 2D**). We investigated the anti-tumor effects of the murine lirilumab analogue aLy49 and avelumab in a human HPV+ TC-1 cervical tumor model (**Supplementary Figure S6E**) and a human HPV-negative C33A cervical tumor model (**Supplementary Figure S6F**). For both models, a set of tumor-bearing mice were simultaneously administered aLy49 (20 mg/kg daily) plus avelumab (2 mg/kg daily) i.p. starting at D7 or D14. aLy49+avelumab administered starting on D7 or D14 reduced TC-1 tumor volume at day 30 and day 38, respectively, and significantly increased 60-day post-implant survival rates (**Figure 2E**). In contrast, aLy49+avelumab had no effect on HPV-negative C33A tumor volumes (**Supplementary Figure S8A**) or survival (**Supplementary Figure S8B**).

### Lirilumab and Avelumab Co-Engagement Upregulates Cytokine and Granzyme B Expression in a NF-κB-Dependent Manner

**Figure 3:**
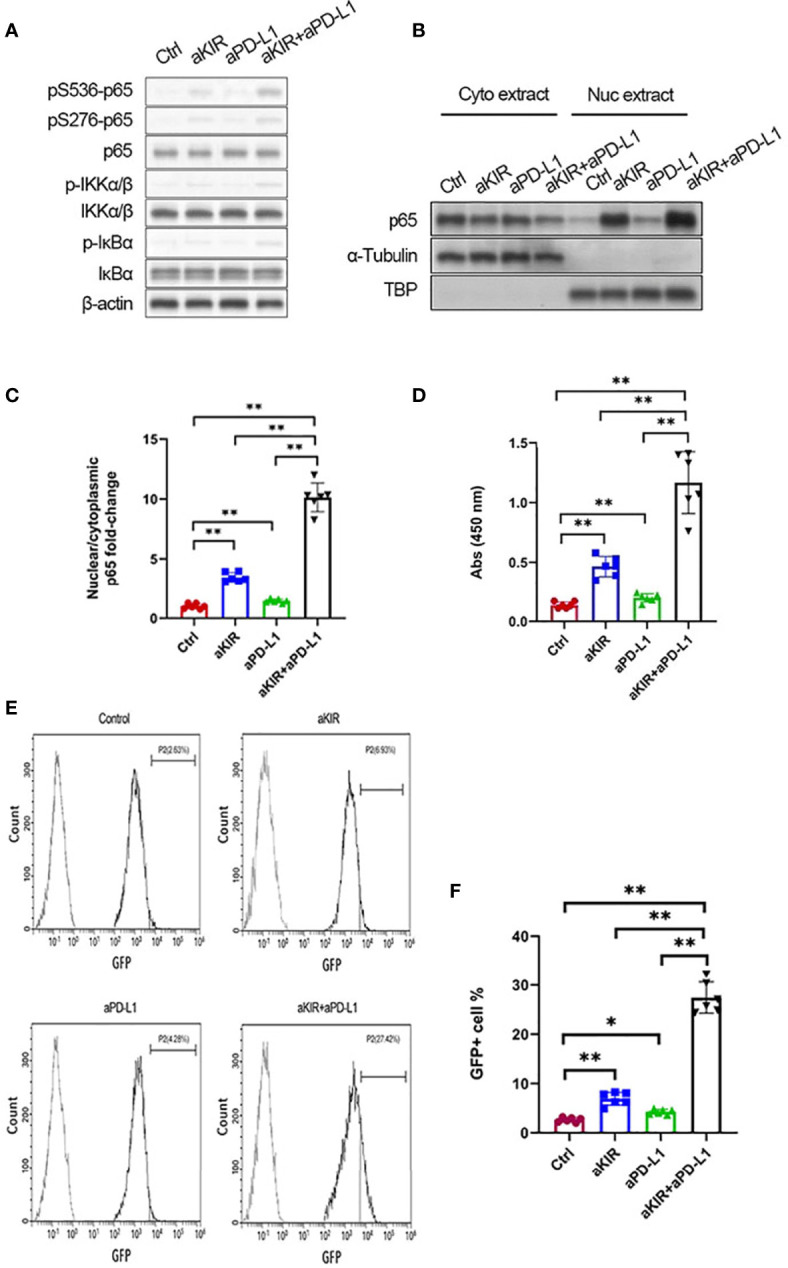
Lirilumab and Avelumab Co-Engagement Disinhibits NF-κB. **(A)** IL-2-primed NK cells were mixed with autologous target cells and stimulated with lirilumab (aKIR) and/or avelumab (aPD-L1) by receptor crosslinking for 4 h. Whole cell lysates were immunoblotted for phospho-S536 p65, phospho-S276 p65, total p65, phospho-S176/180 IKKα/β, total IKKα/β, phospho-IκBα, total IκBα, and β-actin. **(B, C)** IL-2-primed NK cells were mixed with autologous target cells and stimulated with plate-immobilized aKIR and/or aPD-L1 for 1 h. Cytoplasmic and nuclear extracts were immunoblotted with anti-p65 antibody. The ratios of normalized nuclear p65:normalized cytoplasmic p65 are reported. **(D)** Nuclear extracts collected as in **(B)** were added to a 96-well plate immobilized with the consensus NF-kB-binding sequence. p65 bound to the oligonucleotide was measured by colorimetric assay. **(E, F)** IL-2-primed NK cells transduced with a κB-GFP reporter construct were mixed with autologous target cells and stimulated with plate-immobilized aKIR and/or aPD-L1 for 6 h. GFP expression in NK-κB-GFP cells was analyzed by flow cytometry. *P < 0.05, **P < 0.01 [one-way ANOVA]. *n=*3 biological replicates×3 technical replicates.

The NF-κB pathway has been identified as a key downstream signaling pathway of NK cell surface receptors (18). We hypothesized that lirilumab and avelumab may impact NF-κB pathway disinhibition in NK cells. IL-2-primed NK cells mixed with autologous target cells and stimulated with lirilumab or avelumab by receptor crosslinking displayed upregulation in p65, IKKα/β, and IκBα phosphorylation (**Figure 3A** and **Supplementary Figures S9A–D**). Lirilumab+avelumab enhanced these effects compared to either monotherapy (**Figure 3A** and **Supplementary Figures S9A–D**). IL-2-primed NK cells mixed with autologous target cells and stimulated with plate-immobilized lirilumab or avelumab displayed heightened nuclear translocation of p65 (**Figures 3B, C**) as well as higher p65 binding to the consensus NF-kB-binding sequence (**Figure 3D**). Lirilumab+avelumab enhanced these effects compared to either monotherapy (**Figures 3B–D**). IL-2-primed NK cells transduced with a κB-GFP reporter construct mixed with autologous target cells and stimulated with plate-immobilized lirilumab or avelumab displayed higher GFP expression by flow cytometry, an effect potentiated by lirilumab+avelumab (**Figures 3E, F**).

**Figure 4:**
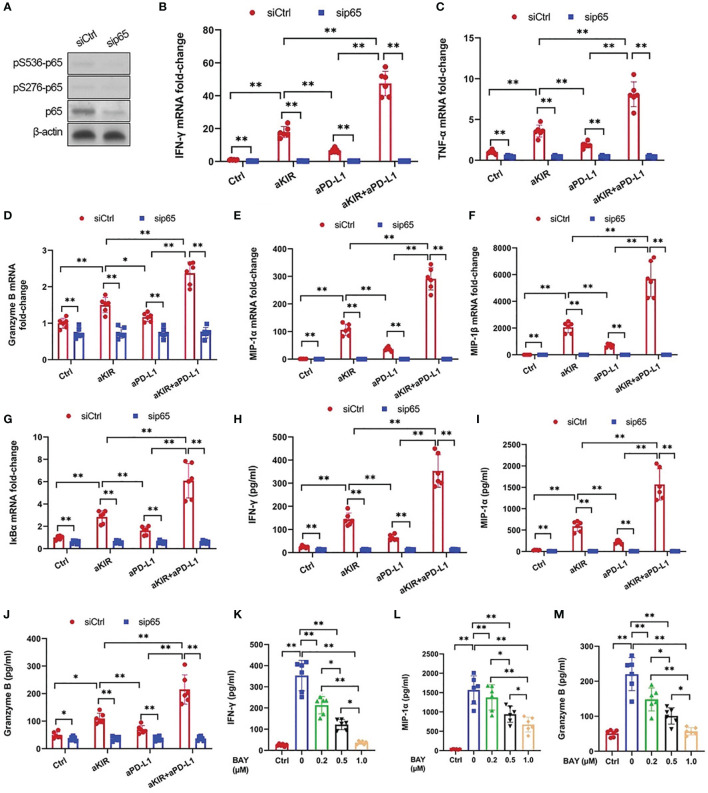
Lirilumab and Avelumab Co-Engagement Upregulates Cytokine and Granzyme B Expression in a NF-κB-Dependent Manner. **(A)** NK cells were transfected with 300 pmol control siRNA (siCtrl) or anti-p65 siRNA (sip65) for 24 h. Cells were rested for another 24 h, and lysates were immunoblotted for p65 and β-actin. **(B-G)** IL-2-primed NK cells transfected with siCtrl or sip65 were mixed with autologous target cells and stimulated with aKIR and/or aPD-L1 for 3 h. Relative mRNA levels of **(B)** IFN-γ, **(C)** TNF-α, **(D)** granzyme B, **(E)** MIP-1α, **(F)** MIP-1β, and **(G)** IκBα were determined by real-time PCR and normalized to β-actin mRNA expression. **(H-J)** Transfected IL-2-primed NK cells were mixed with autologous target cells and stimulated as in **(B-G)** for **(H, I)** 8 h or **(J)** 2 h. **(H)** IFN-γ, **(I)** MIP-1α, and **(J)** Granzyme B were measured by ELISA. **(K–M)** IL-2-primed NK cells were pretreated with the NF-kB inhibitor BAY11-7082 (BAY) at the indicated doses for 1 h, mixed with autologous target cells, and stimulated with both aKIR and aPD-L1 for **(K, L)** 8 h or **(M)** 2 h. **(K)** IFN-γ, **(L)** MIP-1α, and **(M)** Granzyme B were measured by ELISA. *P < 0.05, **P < 0.01 [**(B–J)** two-way ANOVA (mAb factor × transfection factor), **(K–M)** one-way ANOVA]. *n=*3 biological replicates×3 technical replicates.

As lirilumab and avelumab stimulate NF-κB pathway disinhibition in NK cells, we hypothesized that these mAbs would also promote downstream cytokine and granzyme B expression in a NF-κB-dependent manner (18). Therefore, we conducted experiments using p65 silencing in IL-2-primed NK cells mixed with autologous target cells (**Figure 4A**). NK cells stimulated with lirilumab or avelumab displayed higher mRNA expression of several key cytokines, granzyme B, and IκBα, effects potentiated by lirilumab+avelumab (**Figures 4B–G**). NK cells mixed with autologous target cells and stimulated with lirilumab or avelumab showed increased secretion of IFN-γ, MIP-1α, and granzyme B, effects potentiated by lirilumab+avelumab (**Figures 4H–J**). p65 silencing abrogated the mAb-induced gains in cytokine and granzyme B secretion (**Figures 4H–J**). Confirming our results, IL-2-primed NK cells pretreated with various doses of the NF-kB inhibitor BAY11-7082 mixed with autologous target cells and then stimulated with lirilumab+avelumab displayed dose-dependent reductions in IFN-γ, MIP-1α, and granzyme B secretion (**Figures 4K–M**).

### NF-κB Disinhibition by Lirilumab and Avelumab Co-Engagement Is Vav1-Dependent

**Figure 5:**
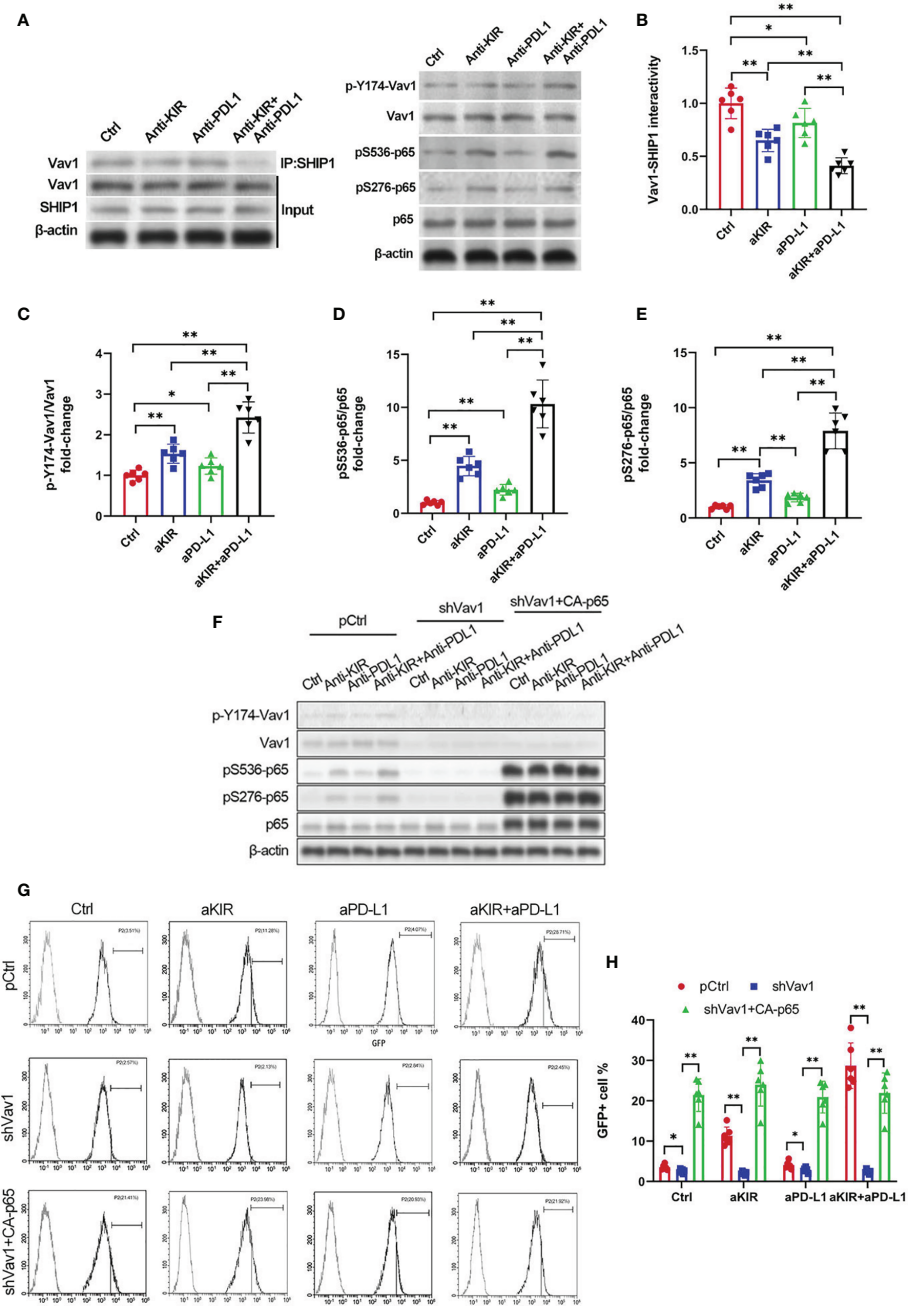
NF-κB Disinhibition by Lirilumab and Avelumab Co-Engagement is Vav1-Dependent. **(A)** IL-2-primed NK cells were mixed with autologous target cells and stimulated with lirilumab (aKIR) and/or avelumab (aPD-L1) by receptor crosslinking for 4 h. Whole cell lysates underwent co-immunoprecipitation for Vav1-SHIP1 binding using an anti-SHIP1 antibody (left) or were immunoblotted for phospho-Y174 Vav1, total Vav1, phospho-S536 p65, phospho-S276 p65, total p65, and β-actin (right). **(B-E)** Normalized intensities of **(B)** Vav1-SHIP1 interactivity, **(C)** phospho-Y174 Vav1, **(D)** phospho-S536 p65, and **(E)** phospho-S276 p65 relative to their total counterparts are reported. **(F)** IL-2-primed NK cells transfected with pCtrl, shVav1, and shVav1+CA-p65 were mixed with autologous target cells and stimulated as in **(A)** for 4 h. Lysates from isolated NK cells were immunoblotted for phospho-Y174 Vav1, total Vav1, phospho-S536 p65, phospho-S276 p65, total p65, and β-actin. **(G, H)** Transfected IL-2-primed NK-κB-GFP cells were mixed with autologous target cells and stimulated with plate-immobilized aKIR and/or aPD-L1 for 6 h. GFP expression in the isolated reporter NK cells was analyzed by flow cytometry. *P < 0.05, **P < 0.01 [**(B–E)** one-way ANOVA or **(H)** two-way ANOVA (mAb factor × transfection factor)]. *n=*3 biological replicates×3 technical replicates.

Disinhibition of NK cells is theorized to involve the integration of multiple receptor-driven pathways at the level of the signal transducer Vav1 (18). Both lirilumab and avelumab negatively regulate immunoreceptor tyrosine-based inhibitory motif (ITIM)’s recruitment of Src homology region 2 domain-containing phosphatase (SHIP, SHP) (24–26), a phosphatase which directly dephosphorylates Vav1 and suppresses downstream NF-κB signaling (27). Therefore, we hypothesized that lirilumab’s and avelumab’s effects upon NF-κB disinhibition may operate through the SHIP/Vav1 axis. Confirming this, IL-2-primed NK cells were mixed with autologous target cells and stimulated with lirilumab or avelumab by receptor crosslinking displayed downregulation of Vav1-SHIP1 interactivity coupled with upregulation of p-Vav1, p-S536 p65, and p-S276 p65 (**Figures 5A–E**). Lirilumab+avelumab enhanced these effects compared to either monotherapy (**Figures 5A–E**).

**Figure 6:**
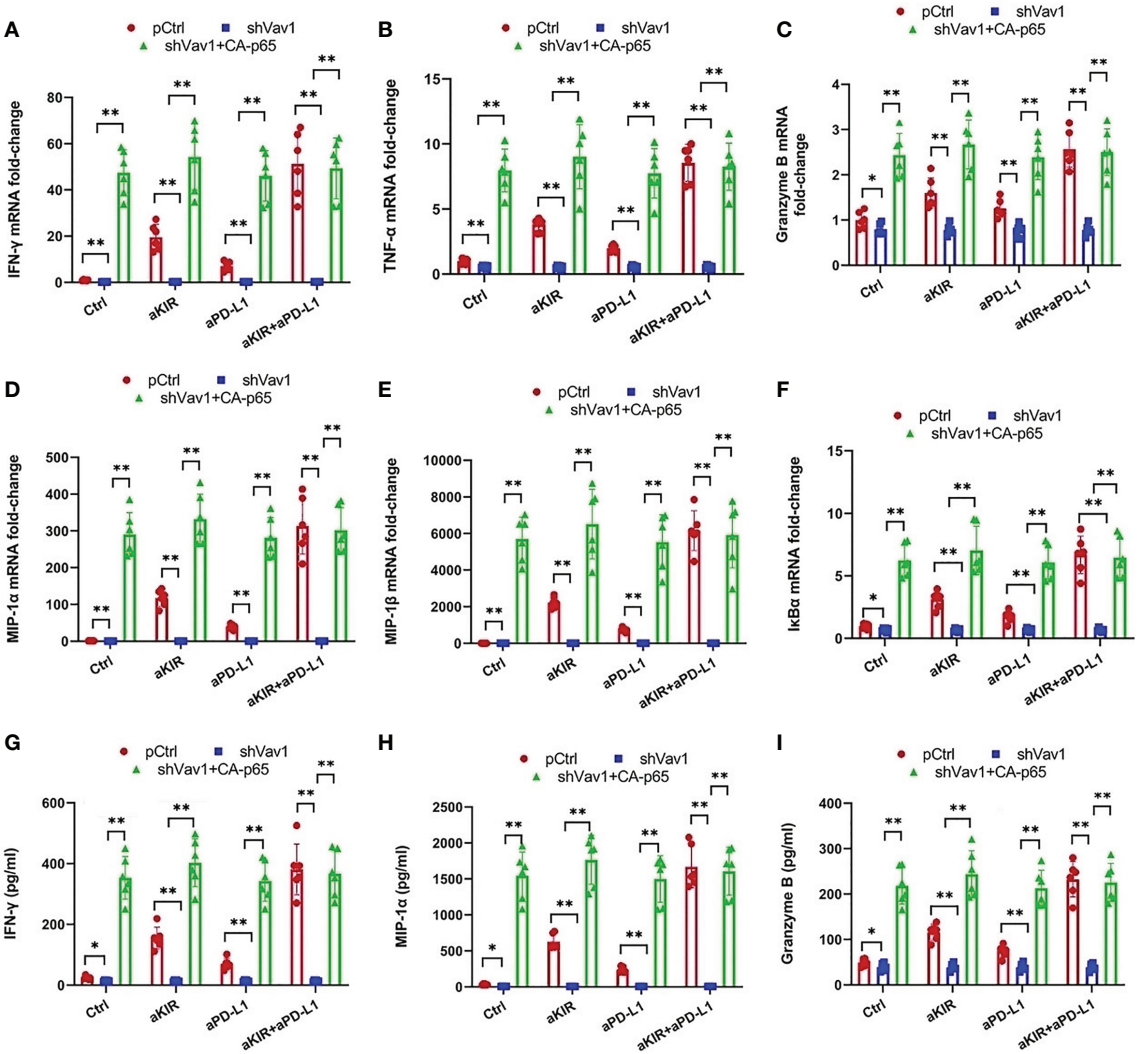
Cytokine and Granzyme B Upregulation by Lirilumab and Avelumab Co-Engagement is Vav1-Dependent. **(A-F)** Transfected IL-2-primed NK cells were mixed with autologous target cells and stimulated with aKIR and/or aPD-L1 for 3 h. Relative mRNA levels of **(A)** IFN-γ, **(B)** TNF-α, **(C)** granzyme B, **(D)** MIP-1α, **(E)** MIP-1β, and **(F)** IκBα were determined by real-time PCR and normalized to β-actin mRNA expression. **(G-I)** Transfected IL-2-primed NK cells were mixed with autologous target cells and stimulated as in **(A-F)** for **(G, H)** 8 h or **(I)** 2 h. **(G)** IFN-γ, **(H)** MIP-1α, and **(I)** Granzyme B were measured by ELISA. *P < 0.05, **P < 0.01 [two-way ANOVA (mAb factor × transfection factor)]. n=3 biological replicates×3 technical replicates.

To establish the role of Vav1 in NF-κB disinhibition by lirilumab and avelumab, we constructed three groups of NK cells by plasmid electroporation: empty plasmid control (pCtrl), anti-Vav1 shRNA (shVav1), and shVav1+constitutively-active p65 phosphomutant p-S536D/S276D p65 (shVav1+CA-p65). IL-2-primed NK cells transfected with shVav1 displayed effective knockdown of phospho- and total Vav1 while those transfected with CA-p65 displayed overexpression of p-S536 p65 and p-S276 p65 (**Figure 5F** and **Supplementary Figures S10A–C**). These Vav1-silenced cells, when mixed with autologous target cells and stimulated with lirilumab and/or avelumab by receptor crosslinking, displayed downregulation in p65 phosphorylation, which was rescued by the addition of CA-p65 (**Figure 5F** and **Supplementary Figures S10A–C**). Vav1-silenced NK-κB-GFP cells mixed with autologous target cells and stimulated with plate-immobilized lirilumab and/or avelumab displayed downregulation in κB-GFP expression, which was rescued by the addition of CA-p65 (**Figures 5G, H**). Similar to p65 silencing, Vav1 silencing abrogated the mAb-induced gains in cytokine, granzyme B, and IκBα expression (**Figures 6A–I**). Notably, these effects were rescued by the addition of CA-p65 (**Figures 6A–I**).

## Discussion

HPV+ cervical cancer patients display cancer cell upregulation of the surface MHC class-I molecule HLA-C as well as the corresponding NK cell surface markers KIR2DL2 and KIR2DL3 (13). HPV+ cervical cancer cells maintain NK cell inhibition *via* HLA-C/KIR2DL2 and HLA-C/KIR2DL3 interactions (13). Lirilumab acts by binding to several human inhibitory KIRs (e.g., KIR2DL1, -L2, -L3, -S1, and -S2), thereby interfering with HLA/KIR interactions (28). This interference by lirilumab blocks KIR ITIM’s recruitment of SHIP, disinhibiting NK cells and promoting their lytic activity against solid tumor cells (24, 25). Consistently, here we found that lirilumab disinhibited NK cells and promoted NK cell-mediated lysis of HPV+ cervical cancer cells. Moreover, a murine lirilumab analogue reduced tumor volumes in a syngeneic murine HPV+ cervical tumor model and a HPV-positive TC-1 cervical tumor model. Our findings concord with similar immunotherapeutic approaches in murine models of B-cell lymphoma (14) and multiple myeloma (17). Notably, KIR blockade by another anti-KIR IgG4 mAb IPH2101 (1-7F9) has been shown to induce endogenous NK cell-mediated lysis of HLA-C-expressing tumor cells (29, 30). However, it remains to be determined whether lirilumab displays a similar effect on endogenous NK cell-mediated lysis of target cells.

HPV has been associated with PD-L1 upregulation in dysplastic and malignant cervical cancer cells as well as PD-1 upregulation on CD8+ cells recruited to the area of HPV infection (6). PD-L1 and PD-1 expression have been positively correlated with HPV+ status, CIN grade elevation, and tumor metastasis (31), and survival outcomes are worse in squamous cell carcinoma tumors with diffuse PD-L1 expression (32). Avelumab acts by binding to PD-L1, thereby interfering with PD-L1/PD-1 interactions (33). Similar to lirilumab, this interference by avelumab blocks PD-1 ITIM’s recruitment of SHIP, disinhibiting NK cells and promoting their lytic activity against solid tumor cells (33). Consistently, here we found that avelumab disinhibited NK cells and promoted NK cell-mediated lysis of HPV+ cervical cancer cells. Moreover, a murine avelumab analogue reduced tumor volumes in a syngeneic murine HPV+ cervical tumor model and a HPV+ TC-1 cervical tumor model. Our findings concord with successful results for PD-L1/PD-1 blockade across a broad range of solid tumor types, including head and neck squamous cell carcinoma (34), melanoma (35), and non-small cell lung cancer (36).

We also discovered that the combination of lirilumab+avelumab enhanced their individual effects, both *in vitro* and *in vivo*. Moreover, this mAb combination increased 60-day post-implant survival rates in the HPV+ murine models. Notably, aLy49+aPdl1 enhanced intratumoral CD107a+ NK cell infiltration and cytolysis at day 30 in our syngeneic murine HPV+ cervical tumor model. This phenomenon may be attributed to the fact that this mAb combination targets two separate but converging inhibitory NK cell pathways – lirilumab targeting the HLA-C/KIR/SHIP pathway and avelumab targeting the PD-L1/PD-1/SHIP pathway (24–26). ITIM-activated SHIP has been shown to dephosphorylate the signal integrator Vav1 and inhibit downstream NF-κB signaling (27). Indeed, here we found that lirilumab or avelumab by receptor crosslinking displayed downregulation of Vav1-SHIP1 interactivity coupled with upregulation of Vav1 phosphorylation and p65 phosphorylation. Moreover, lirilumab+avelumab enhanced this NF-κB pathway disinhibition compared to either monotherapy. Notably, Vav1 silencing eliminated this disinhibition of NF-κB signaling by lirilumab and/or avelumab in NK cells. These findings support Kwon et al.’s postulation that Vav1 represents a molecular checkpoint that integrates upstream signals to properly control NF-κB pathway disinhibition, thereby ensuring appropriate NK cell responses against target cells (18).

In conclusion, this study presents preclinical evidence supporting a novel approach to enhancing NK cell lysis against autologous HPV+ cervical cancer cells through combining lirilumab and avelumab immunotherapy. These results provide a strong rationale for combining anti-KIR and anti-PD-L1 mAb therapies in HPV+ cervical cancers in order to “remove the brakes” from NK cells and further enhance their lytic capacity.

## Data Availability Statement

The original contributions presented in the study are included in the article/**Supplementary Material**. Further inquiries can be directed to the corresponding authors.

## Ethics Statement

The studies involving human participants were reviewed and approved by the Ethics Review Committee of the First Affiliated Hospital of Bengbu Medical College (approval no. 2020-088). The patients/participants provided their written informed consent to participate in this study. The animal study was reviewed and approved by the Ethics Review Committee of the First Affiliated Hospital of Bengbu Medical College (approval no. 2020-088).

## Author Contributions

HL, FC, XW, and NW conceived the experiment. HL, FC, SZ, JL, YZ, ML, SM conducted the experiment. All the authors analyzed and discussed the results. NW, XW guided the work. HL wrote the first draft. NW, HW, and XW revised the first draft. All the authors approved the final version of the manuscript.

## Funding

This work was supported by the Key Program of Natural Science Research of Higher Education of Anhui Province (grant KJ2019A0346) and the 512 Talent Cultivation Project of Bengbu Medical College (grant by51201108).

## Conflict of Interest

The authors declare that the research was conducted in the absence of any commercial or financial relationships that could be construed as a potential conflict of interest.

## Publisher’s Note

All claims expressed in this article are solely those of the authors and do not necessarily represent those of their affiliated organizations, or those of the publisher, the editors and the reviewers. Any product that may be evaluated in this article, or claim that may be made by its manufacturer, is not guaranteed or endorsed by the publisher.
