# Supplementary material for: Lirilumab and Avelumab Enhance Anti-HPV+ Cervical Cancer Activity of Natural Killer Cells via Vav1-Dependent NF-κB Disinhibition
Source: Front Oncol. 2022 Jan 31;12:747482. doi: 10.3389/fonc.2022.747482 (PMC8841689; doi:10.3389/fonc.2022.747482)
Supplement: Supplementary file 1 [file DataSheet_1.pdf]

SUPPLEMENTARY INFORMATION FOR

Lirilumab and Avelumab Enhance Anti-HPV+ Cervical Cancer Activity of Natural Killer Cells via Vav1-Dependent NF-κB Disinhibition

SUPPLEMENTARY METHODS

Patient Sample Collection

Cervical biopsy samples and blood samples were collected from stage I-II HPV16-positive cervical squamous cell carcinoma patients (*n*=24). Biopsy samples and blood samples were also obtained from age-matched healthy HPV-negative female donors (*n*=24) with no evidence of pre-malignant/malignant cervical disease. Malignant cervical squamous cells and normal cervical squamous cells were isolated from the biopsy samples of the cancer patients and healthy donors, respectively (1). Peripheral blood mononuclear cells (PBMCs) were isolated from blood samples via density-gradient centrifugation (LSM Lymphocyte Separation Medium, MP Biomedicals, Santa Ana, CA). The cells were frozen down at -80°C until later use.

Antibodies

The monoclonal antibody (mAb) lirilumab was obtained from Innate Pharma (Marseille, France). The mAb avelumab was obtained from EMD Serono (Billerica, MA). The anti-mouse F(ab')2 IgG for receptor crosslinking was purchased from Jackson ImmunoResearch (West Grove, PA). The murine mAb analogue for lirilumab (anti-Ly49C and I, aLy49) was obtained from Pharmingen (San Diego, CA) (2). The murine mAb analogue for avelumab (anti-mouse Pdl1, aPdl1) was obtained from Bio-XCell (clone 10F.9G2) (3).

The conjugated antibodies employed for flow cytometry were sourced as follows: isotype control rabbit IgG-Alexa Fluor 488 (DA1E; Cell Signaling); PE-labeled anti-lirilumab-PE (Innate Pharma); anti-HLA-C-DyLight 488 (DT-9, Novus Biologicals); anti-avelumab-PE (EMD Serono); anti-PD1-Alexa Fluor 647 (EH12.1), anti-α-CD3-PerCP (SK7), anti-α-CD56-PE (NCAM16.2), and anti-α-CD107a-FITC (H4A3) (BD Biosciences, San Jose, CA).

Antibodies for the murine tumor model and co-immunoprecipitation/immunoblotting were sourced as follows: isotype control mouse IgG (MOPC-21; Sigma); pS32/36 IκBα (39A1413) and β-actin (C4) (BD Biosciences); α-tubulin (GT114; GeneTex); pS276 p65 (ab30623; Abcam); p65 (F-6), IκBα (C-21), and SHIP1 (P1C1; Santa Cruz); Vav1 (R775), pS536 p65 (93H1), pS176/180 IKKα/β (16A6), and TBP (44059; Cell Signaling).

NK Cell Isolation

Primary human NK cells were isolated from blood-derived PBMCs via lymphocyte flow cytometric gating as previously described (4). Briefly, thawed PBMCs were incubated with anti-α-CD3-PerCP and anti-α-CD56-PE antibodies or isotypic controls at 4°C for 30 minutes, washed, and then sorted by FACSCalibur flow cytometer (BD Biosciences). NK cells were isolated by CD3-/CD56+ gating using lymphocyte FSC/SSC parameters, followed by doublet elimination based on FSC-A/FSC-H parameters and dead cell removal (Live/Dead Aquadead kit, Invitrogen, Life Technologies, Grand Island, NY). The final cell composition was >97% CD3-CD56^{+} according to flow cytometry.

Antibody Binding and Antigen Expression Assays

Antibody binding and antigen expression assays were performed as previously described with minor modifications (5). Briefly, cells were stained with the appropriate conjugated antibodies, incubated for 15 minutes at 4°C, and finally washed with MACS buffer. Then, cells were sorted by a FACSCalibur flow cytometer (BD Biosciences) and analyzed with FlowJo 7.6.1 (TreeStar, Ashland, OR). Validation of lirilumab binding and PD-1 antigen expression on blood-derived NK cells as well as avelumab binding and HLA-C antigen expression on tumor-derived cervical cancer cells is provided in Supplementary Fig. S1 (Supplementary Information).

Cell Mixing

Cell mixing was performed as previously described (6). Briefly, following their respective treatments, NK cells and autologous target cells were separately chilled on ice. Cells were then mixed at the indicated effector-to-target (E:T) ratios on ice, incubated for 10 min on ice, and then incubated together for the indicated times at the indicated temperatures.

Europium-Release Cytotoxicity Assays

Cytotoxicity was analyzed by a europium-release assay (DELFIA EuTDA Cytotoxicity Reagents kit; PerkinElmer, Waltham, MA) as previously described with minor modifications (5). Briefly, NK cells were incubated with lirilumab or DMSO (control) for 24 h at 4°C, while autologous target cells were incubated with avelumab or DMSO (control) for 24 h at 4°C. Target cells were then stained with DELFIA BATDA at 37°C. NK cells were then mixed with DELFIA BATDA-labeled autologous target cells and incubated for 30 min at 37°C. Specific cytotoxicity was calculated with the following formula: % cytotoxicity = 100 × (experimental release - spontaneous release) ÷ (maximal release - spontaneous release).

ELISPOT Assays

ELISPOT assays were performed with MultiScreen 96-well plates (Millipore, Billerica, MA) to measure IFN-γ and granzyme B production as previously described (5). Spot forming units (SFU) per well were counted with an Immunospot Imaging Analyzer (Cellular Technology, Shaker Heights, OH).

NK Cell CD107a Expression by Flow Cytometry

NK cell CD107a expression was assayed as previously described with minor modifications (5). Briefly, NK cells were incubated with lirilumab or DMSO (control) for 24 h. Autologous target cells were incubated with avelumab or DMSO (control) for 24 h. Then, in the presence of anti-CD107a-FITC antibody, NK cells were mixed with autologous target cells at a 50 E:T ratio. Cells were then spun down at 30 g for 3 min, incubated for 5 h at 37°C, and spun down again at 30 g for 3 min. The pellets were immediately resuspended in FACS buffer and stained with anti-CD107a-PE antibody in the dark at 4°C for 30 min. The NK cells were gated by forward scatter/side scatter (FACSCalibur flow cytometer, BD Biosciences), and NK cell CD107a expression was analyzed with FlowJo 7.6.1 (TreeStar).

Construction of HPV+ Murine Cervical Cancer Cell Line

The Kunming mouse-derived cervical carcinoma cell line U14 (RRID: CVCL_9U56) was obtained from the Chinese Academy of Medical Sciences Tumor Cell Bank (Beijing, China) and were passaged in our lab within one month of receipt. The methods of establishment and characterization of the U14 cell

line by the Chinese Academy of Medical Sciences -- including morphological analysis, growth analysis, immunohistochemistry, cell cycle analysis, chromosomal analysis, and *in vivo* transplantation analysis -- have been fully reported elsewhere (7). U14 cultures were confirmed to be free of *Mycoplasma* and then maintained at 37° in 5% CO_2 in RPMI-1640 medium with 10% fetal calf serum (FCS), 100 U/ml penicillin, 100 mg/ml streptomycin, and 2 mM L-glutamine (Hyclone, GE Healthcare Life Sciences Logan, UT). The human HPV16 E6 and E7 genes were then transfected into the U14 cell line, and successful E6+/E7+ double-transfected clones were selected for further culture as previously described (8).

Syngeneic Murine Tumor Model

A syngeneic murine HPV+ cervical tumor model was constructed as previously described with minor modifications (9). Briefly, female specified pathogen-free (SPF) Kunming mice (8-12 weeks old) were obtained from our university's animal center. Syngeneic HPV+ U14 cells (2×10^5 per subject) were subcutaneously (s.c.) implanted into the mice subjects' left flanks on days 7, 14, or 25 prior to immunization. Subjects were randomly assigned into four groups (*n*=12 per group) and intraperitoneally (i.p.) injected using a 30G needle with either (i) control IgG (0.5 mg/kg daily), (ii) IgG plus aLy49 (40 or 20 mg/kg daily), (iii) IgG plus aPdl1 (4 or 2 mg/kg daily), or (iv) aLy49 plus aPdl1 daily. To assess tumor growth, individual tumors were measured with electronic calipers three times per week. Tumor volumes were calculated with the following formula: $0.5 \times \text{length} \times \text{width}^2$. After sacrifice, the harvested tumors were immediately frozen down at -80°C until later analysis.

Scoring of Immune Cell Infiltration within Murine Tumors

To comparatively assess the levels of immune cell infiltration within the harvested murine tumors, we employed a previously-validated computational method that uses single-sample gene set enrichment analysis (ssGSEA) (10) to analyze immune cell-specific gene signatures within 30 day-old tumor samples. Briefly, ssGSEA produces an overexpression score for a particular immune cell-specific gene signature by comparing the ranks of the immune cell-specific signature genes against that of all other transcriptome genes (11). We characterized immune cell infiltration within the murine tumors via

ssGSEA with immune cell-specific gene signatures for key innate and adaptive immunity cells. As previously defined, the T-cell infiltration score (TIS) was the average of the standardized scores for CD8+ T-cells, Treg cells, central and effector memory T-cells, Th1, Th2, and Th17 cells, while the overall immune infiltration score (IIS) was the average of the standardized scores for neutrophils, eosinophils, macrophages, mast cells, dendritic cells, CD107a+ NK cells, cytotoxic cells, B-cells, and all T-cell subsets except T-gamma delta and T-follicular helpers (12). The cytolytic score was calculated using the mRNA levels of three key cytolytic genes (granzyme A, granzyme B, and perforin) as previously described (13).

IL-2 Priming of NK Cells

NK cells were incubated in the presence of IL-2 (200 U/ml) for 36 h and rested for 12 h prior to experimental use.

Receptor-Crosslinking and Plate-Immobilized Antibody Stimulation

Receptor-crosslinking antibody stimulation was performed as previously described with minor modifications (6). IL-2-primed NK cells were treated with an isotype-control antibody or lirilumab (10 μg/ml) at 4°C for 30 min, while autologous target cells were treated with an isotype-control antibody or avelumab (10 μg/ml) at 4°C for 30 min. After rinsing off any unbound antibodies, IL-2-primed NK cells and autologous target cells were then mixed at a 50 E:T ratio and stimulated by crosslinking with anti-mouse F(ab)2 secondary antibody (30 μg/ml) at 37 °C for the indicated times. Thereafter, NK cells were isolated by FACS (FACSCalibur flow cytometer, BD Biosciences).

Plate-immobilized antibody stimulation was performed as previously described with minor modifications (6). Briefly, Costar 96-well EIA/RIA Stripwell plates (Corning, Corning, NY) were coated overnight with an isotype-control antibody, lirilumab, and/or avelumab (10 μg/ml each) at 4°C. IL-2-primed NK cells and autologous target cells were then mixed at a 50 E:T ratio and added onto the coated plates for the indicated times. For some experiments, IL-2-primed NK cells were pretreated with the NF-kB inhibitor BAY11-7082 (BAY) at the indicated doses for 1 h prior to mixing. Thereafter, NK cells were isolated by FACS (FACSCalibur flow cytometer, BD Biosciences).

Co-immunoprecipitation (co-IP)

Isolated NK cells were washed with ice-cold PBS and lysed for 30 min on ice in lysis buffer (1% Triton X-100, 150 mM NaCl, 50 mM Tris-HCl (pH 7.5), 50 mM NaF, 5 mM EDTA, 1 mM NaVO3, 1 mM phenylmethylsulfonyl fluoride (PMSF) and protease inhibitor cocktail [Thermo]). The lysates were centrifuged down (12,000 g, 10 min) and supernatant protein content was measured with a BCA protein assay kit (Pierce). For co-IP, 1 mg protein/ml was mixed with 4 μg anti-SHIP1 antibody or negative control IgG for 3 h. The antibody-antigen complexes were precipitated using protein G-linked Sepharose beads (Pharmacia) for 30 min. The beads were washed twice with lysis buffer, and the resulting co-IP products were subjected to immunoblotting as described below.

Fractionation and Immunoblotting

Isolated NK cells were lysed as described above, and the cytoplasmic and nuclear fractions were isolated using a Nuclear Extract kit (Active Motif, Carlsbad, CA). Protein concentration determination, protein resolution by SDS-PAGE, and PVDF membrane transfer were also performed as previously described (6). Membranes were blocked with skim milk in TBS-T for 1 h, incubated with primary antibodies, and then incubated with HRP-conjugated secondary antibodies. The membranes were finally developed using an enhanced chemiluminescence detection system (Millipore).

NF-κB DNA Binding Assay

NF-κB DNA binding activity was assessed using a Trans-AM enzyme-linked immunosorbent assay (ELISA) kit (Affymetrix-Panomics, Fremont, CA). Briefly, nuclear fractions isolated above were added to Costar 96-well EIA/RIA Stripwell plates (Corning, Corning, NY) immobilized with double-stranded oligonucleotide containing the consensus NF-kB-binding sequence (5'-GGG ACT TTC C-3'). The amount of p65 bound to the oligonucleotide was measured by a colorimetric reaction generated by specific primary antibody in combination with a horseradish peroxidase-conjugated secondary antibody.

NF-κB Reporter Assay

NK reporter cells (NK-κB-GFP) expressing GFP under NF-κB transcription response element control were constructed by transducing NK cells with a lentiviral κB-GFP construct as previously

described (6). Briefly, lentiviral particles were constructed through simultaneously transfecting a 293TN packaging cell line with a pGreenFire-NF-κB-EF1-Puro vector and a pPACKH1 packaging plasmid mix (System Biosciences, Mountain View, CA). NK cells were then transduced with the lentivirus supernatant in the presence of 10 μg/ml polybrene and 200 U/ml recombinant IL-2. Cells were then selected with 1 μg/ml puromycin two days following transduction. Then, transduced NK cells displaying GFP expression upon TNF-α challenge were selected by FACS and cultured. NK-κB-GFP cells and autologous target cells were then mixed and stimulated by plate-immobilized antibodies as described earlier. Thereafter, NK cells were isolated by FACS, and GFP expression was analyzed (FACSCalibur flow cytometer, BD Biosciences).

Supernatant ELISA

IFN-γ, MIP-1α, and Granzyme B release by NK cells after antibody stimulation were assessed by ELISA as previously described with minor modifications (6). Briefly, IL-2-primed NK cells and autologous target cells were mixed and stimulated with plate-immobilized antibodies for 2 h. The supernatants were finally ELISA-assayed for IFN-γ and MIP-1α (RayBiotech, Norcross, GA) as well as granzyme B (eBioscience, San Diego, CA).

Silencing of p65 Expression by Small-Interfering RNA (siRNA)

siRNA experiments were performed using the Amaxa Nucleofector II transfection system (Lonza Group, Basel, Switzerland) as previously described (6). Briefly, 1.5×10^6 NK cells were resuspended in 100 μl Amaxa kit solution for human macrophages, mixed with 300 pmol siRNA and transfected using the X-001 program. An optimally effective siRNA was used to silence NF-κB p65 subunit gene expression (sip65) as previously described (6): 5'-GGA GUA CCC UGA GGC UAU AAC UCG C-3' (sense) and 5'-GCG AGU UAU AGC CUC AGG GUA CUC CAU-3' (antisense). siRNA negative control oligos (siCtrl) were purchased from Dharmacon (Lafayette, CO).

Vav1 Silencing and p65 Phosphomutant Overexpression by Plasmid Electroporation

Plasmid electroporation was performed as previously described (14). Briefly, a small-hairpin RNA (shRNA) sequence against human Vav1 (shVav1; sc-29517-SH, SCBT) without or with the full-length

cDNA of the human p65 phosphomutant (CA-p65) were cloned into the pEGFP-N1 plasmid (BD Biosciences, San Jose, CA) to respectively form the pEGFP-N1-shVav1 and pEGFP-N1-shVav1+CA-p65 plasmids. The empty pEGFP-N1 plasmid was used as a negative control (pCtrl). Plasmid DNA was purified from *E. coli*-transformed cells with the EndoFree Plasmid Maxi kit (Qiagen, Hilden, Germany), and then re-suspended in milliQ H_2O. Light absorption (260-nm) was used to assay DNA concentrations. Plasmid DNA quality was calculated by light absorptions at 260/280 and 260/230 nm.

Aforedescribed IL-2-activated NK cells were electroporated at room temperature using the Neon Transfection System (Thermo Fisher Scientific, Waltham, MA) using the following optimal parameters: 120 μg/ml plasmid, 4×10^7 cells/ml, electroporation first pulse of 1850 V and 20 ms, electroporation second pulse of 500 V and 100 ms, and Buffer O with the 0.1% of buffer CD. After electroporation, NK cells were rested in the presence of IL-2 (200 U/ml) for 24 h prior to experimentation.

Quantitative Real-Time RT-PCR

Quantitative real-time RT-PCR (qRT-PCR) was applied to assess transcript expression as previously described (6). Briefly, total RNA was isolated with a RNeasy Mini kit (Qiagen China Co., Ltd., Shanghai, China), and cDNA was reverse-transcribed with an iScript cDNA synthesis kit (Bio-Rad, Hercules, CA). qRT-PCR was performed with SYBR Green and a CFX96 Thermal Cycler (Bio-Rad). The primer sequences used for the qRT-PCR have been detailed by Kwon et al. (6). mRNA expression was normalized to β-actin transcript expression with the ΔΔCt method.

Statistical Analyses

All data are reported as means and associated standard deviations (SDs) derived from at least three independent experiments. Comparisons between two experimental conditions were analyzed using Student's *t*-test as indicated in the Figure Legends. Comparisons among three or more experimental conditions were analyzed by one-way or two-way ANOVA with a Bonferroni post-hoc correction as indicated in the Figure Legends. Comparisons between Kaplan-Meier survival curves were analyzed using log-rank testing. The statistical significance threshold for all comparisons was set at 0.05.

REFERENCES FOR SUPPLEMENTARY INFORMATION

1. M. Aubele, H. Zitzelsberger, U. Schenck, A. Walch, H. Höfler and M. Werner: Distinct cytogenetic alterations in squamous intraepithelial lesions of the cervix revealed by laser-assisted microdissection and comparative genomic hybridization. *Cancer Cytopathology*, 84(6), 375-379 (1998)

2. C. Y. Koh, B. R. Blazar, T. George, L. A. Welniak, C. M. Capitini, A. Raziuddin, W. J. Murphy and M. Bennett: Augmentation of antitumor effects by NK cell inhibitory receptor blockade in vitro and in vivo. *Blood*, 97(10), 3132-3137 (2001)

3. L. Deng, H. Liang, B. Burnette, M. Beckett, T. Darga, R. R. Weichselbaum and Y.-X. Fu: Irradiation and anti–PD-L1 treatment synergistically promote antitumor immunity in mice. *The Journal of clinical investigation*, 124(2), 687-695 (2014)

4. C. Pasero, G. Gravis, S. Granjeaud, M. Guerin, J. Thomassin-Piana, P. Rocchi, N. Salem, J. Walz, A. Moretta and D. Olive: Highly effective NK cells are associated with good prognosis in patients with metastatic prostate cancer. *Oncotarget*, 6(16), 14360 (2015)

5. D. M. Benson, C. E. Bakan, S. Zhang, S. M. Collins, J. Liang, S. Srivastava, C. C. Hofmeister, Y. Efebera, P. Andre and F. Romagne: IPH2101, a novel anti-inhibitory KIR antibody, and lenalidomide combine to enhance the natural killer cell versus multiple myeloma effect. *Blood*, 118(24), 6387-6391 (2011)

6. H.-J. Kwon, G.-E. Choi, S. Ryu, S. J. Kwon, S. C. Kim, C. Booth, K. E. Nichols and H. S. Kim: Stepwise phosphorylation of p65 promotes NF-κB activation and NK cell responses during target cell recognition. *Nature communications*, 7 (2016)

7. B. Gu, H. Feng, J. Dong, H. Zhang, X. Bian and Y. Liu: The establishment and characterization of a continuous cell line of mouse cervical carcinoma. *Chinese Journal of Clinical Oncology*, 5(1), 44-48 (2008)

8. G. Tao, S. Yang and Q. Lin: Growth behavior and metastatic pattern of cervical cancer U14 transfected with human papillomavirus in inbred mouse. *Hunan yi ke da xue xue bao= Hunan yike daxue xuebao= Bulletin of Hunan Medical University*, 26(6), 515-519 (2001)

9. L.-S. Chang, C.-H. Leng, Y.-C. Yeh, C.-C. Wu, H.-W. Chen, H.-M. Huang and S.-J. Liu: Toll-like receptor 9 agonist enhances anti-tumor immunity and inhibits tumor-associated immunosuppressive cells numbers in a mouse cervical cancer model following recombinant lipoprotein therapy. *Molecular cancer*, 13(1), 60 (2014)

10. Y. Senbabaoglu, A. G. Winer, R. S. Gejman, M. Liu, A. Luna, I. Ostrovnaya, N. Weinhold, W. Lee, S. D. Kaffenberger and Y. B. Chen: The landscape of T cell infiltration in human cancer and its association with antigen presenting gene expression. *bioRxiv*, 025908 (2015)

11. G. Bindea, B. Mlecnik, M. Tosolini, A. Kirilovsky, M. Waldner, A. C. Obenauf, H. Angell, T. Fredriksen, L. Lafontaine and A. Berger: Spatiotemporal dynamics of intratumoral immune cells reveal the immune landscape in human cancer. *Immunity*, 39(4), 782-795 (2013)

12. R. Mandal, Y. Şenbabaoğlu, A. Desrichard, J. J. Havel, M. G. Dalin, N. Riaz, K.-W. Lee, I. Ganly, A. A. Hakimi and T. A. Chan: The head and neck cancer immune landscape and its immunotherapeutic implications. *JCI insight*, 1(17) (2016)

13. M. S. Rooney, S. A. Shukla, C. J. Wu, G. Getz and N. Hacohen: Molecular and genetic properties of tumors associated with local immune cytolytic activity. *Cell*, 160(1-2), 48-61 (2015)

14. T. Ingegnere, F. R. Mariotti, A. Pelosi, C. Quintarelli, B. De Angelis, N. Tumino, F. Besi, C. Cantoni, F. Locatelli and P. Vacca: Human CAR NK cells: a new non-viral method allowing high efficient transfection and strong tumor cell killing. *Frontiers in immunology*, 10, 957 (2019)

SUPPLEMENTARY TABLES

Supplementary Table S1. Estimated Tumor Volumes at Initiation of Therapy

**Model (day of therapy initiation)**	**Estimated tumor volumes (mean ± SD, mm^3)**	**Significant difference between cohorts?**
Fig. 2A (D7)	Control: 12.96 ± 0.74[*]	NS
	aLy49 (20 mg/kg): 12.81 ± 0.86[†]	
	aLy49 (40 mg/kg): 12.62 ± 1.04	
Fig. 2B (D7)	Control: 12.96 ± 0.74[*]	NS
	aPdl1 (2 mg/kg): 12.47 ± 0.73[‡]	
	aPdl1 (4 mg/kg): 12.32 ± 1.01	
Fig. 2C (D7)	Control: 12.96 ± 0.74[*]	NS
	aLy49 (20 mg/kg): 12.81 ± 0.86[†]	
	aPdl1 (2 mg/kg): 12.47 ± 0.73[‡]	
	aLy49+aPdl1: 13.01 ± 0.97[§]	
Fig. 2D (D7)	Control: 12.96 ± 0.74[*]	
	aLy49+aPdl1: 13.01 ± 0.97[§]	
Fig. 2D (D14)	Control: 51.82 ± 4.45	NS
	aLy49+aPdl1: 52.90 ± 6.28	
Fig. 2E (D7)	Control: 38.38 ± 4.16	NS
	aLy49+aPD-L1: 36.64 ± 2.68	
Fig. 2E (D14)	Control: 131.58 ± 15.84	NS
	aLy49+aPD-L1: 134.86 ± 8.23	
Supp. Fig. 8A (D7)	Control: 20.16 ± 1.19	NS
	aLy49+aPD-L1: 20.23 ± 2.86	

[*†‡§]These data represent the same cohorts.

Abbreviations: SD, standard deviation; D7, day 7; D14, day 14; NS, non-significant (P>0.05 by t-test or one-way ANOVA).

SUPPLEMENTARY FIGURES

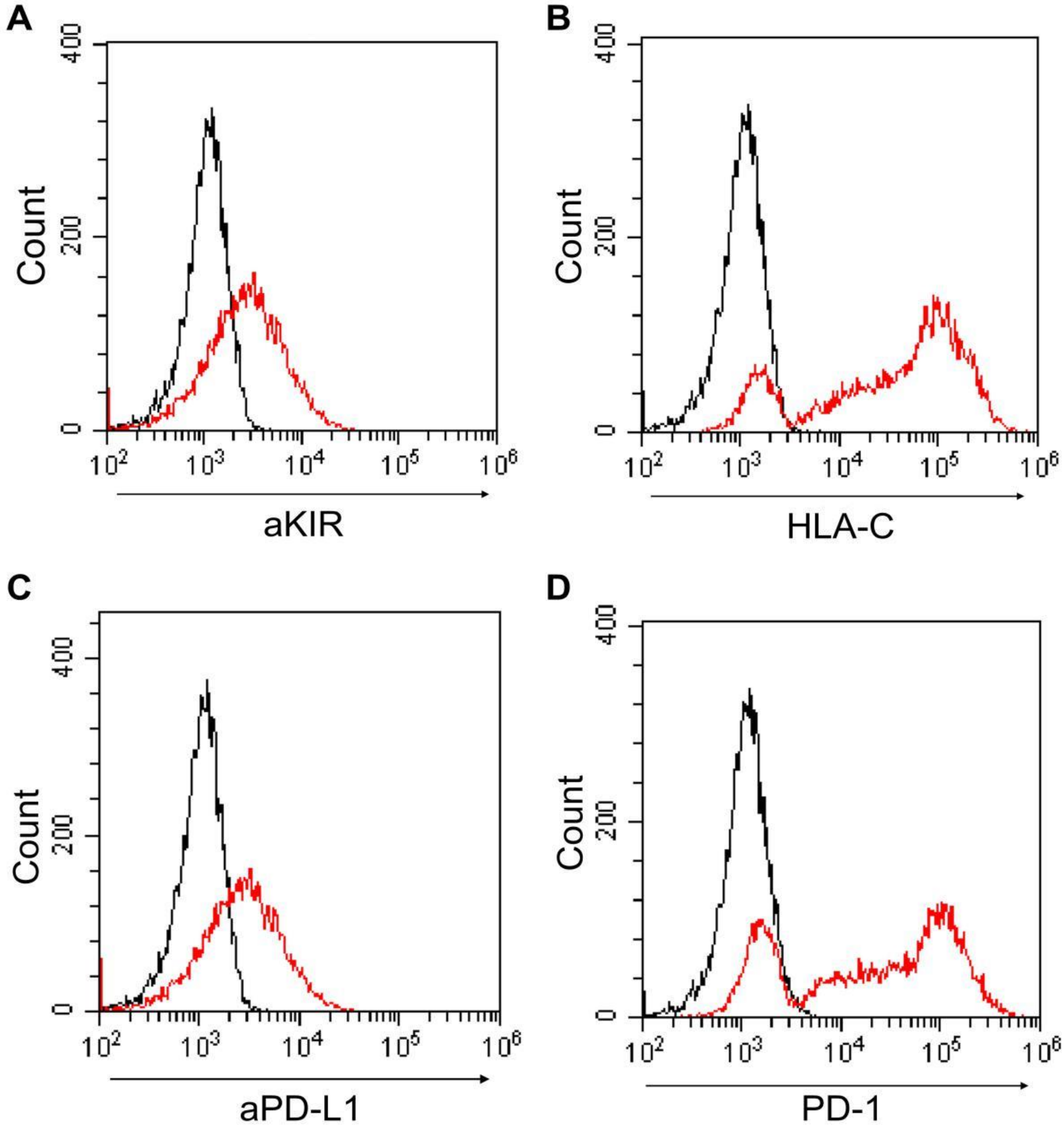

Supplementary Fig. S1. Validation of Antibody Binding and Antigen Surface Expression. Representative flow cytometric images validating (A) positive lirilumab (aKIR) binding on human blood-derived NK cells and (B) positive HLA-C expression on human tumor-derived cervical cancer cells. Representative flow cytometric images validating (C) positive avelumab (aPD-L1) binding to human tumor-derived cervical cancer cells and (D) positive PD-1 expression on human blood-derived NK cells. Isotype control peaks depicted in black. All *in vitro* experiments: n=3 biological replicates × 3 technical replicates.

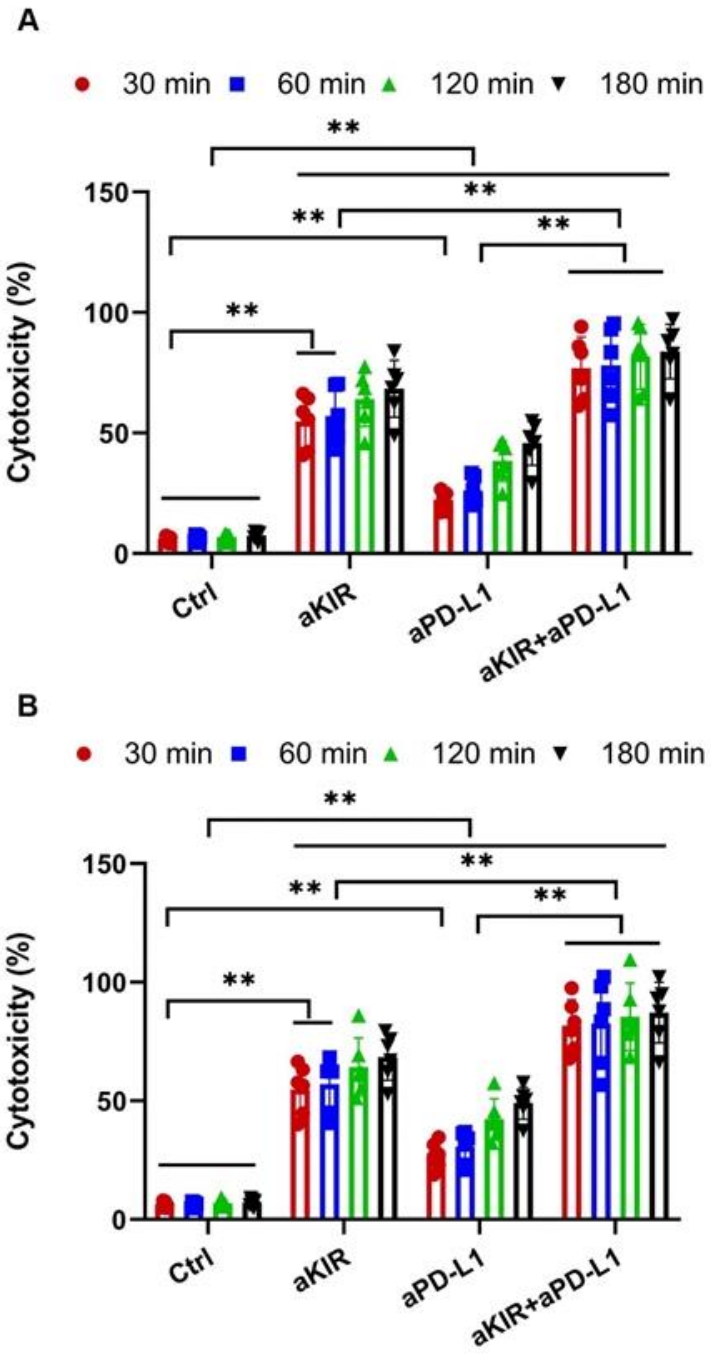

Supplementary Fig. S2. Lirilumab and Avelumab Promote NK Cell Cytotoxicity against HPV+ Cervical Cancer Cells. Blood-derived NK cells and autologous biopsy-derived malignant squamous cells from HPV+ cervical cancer patients (n=24) were incubated with lirilumab (aKIR) and/or avelumab (aPD-L1), respectively, for 24 hours and then co-cultured for various time points. (A, B) aKIR (20 µg/ml) plus aPD-L1 (2.0 µg/ml) augmented NK cell-mediated lysis of cancer cells measured by a target-based, europium-release cytotoxicity assay at E:T ratios of (A) 25:1 and (B) 50:1 over various time points. *P<0.05, **P<0.01 [two-way ANOVA; mAb factor × time factor]. All *in vitro* experiments: n=3 biological replicates × 3 technical replicates.

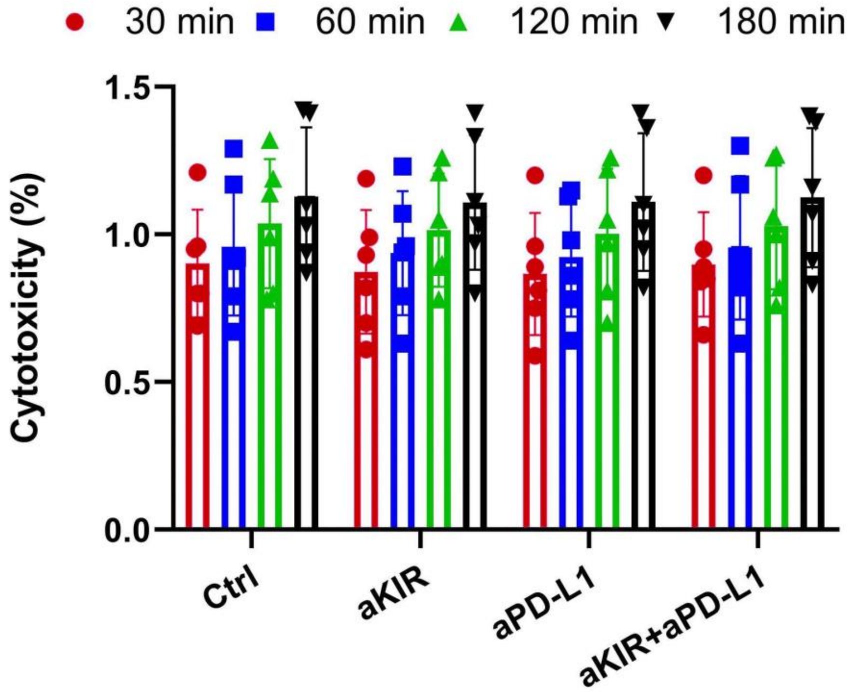

Supplementary Fig. S3. Lirilumab nor Avelumab Promote NK Cell Cytotoxicity against Normal Cervical Squamous Cells. Neither aKIR (20 µg/ml), aPD-L1 (2.0 µg/ml), nor aKIR+aPD-L1 enhanced NK cell-mediated cytotoxicity against normal cervical squamous cells over various time points. *P<0.05, **P<0.01 [two-way ANOVA; mAb factor × time factor]. n=3 biological replicates × 3 technical replicates.

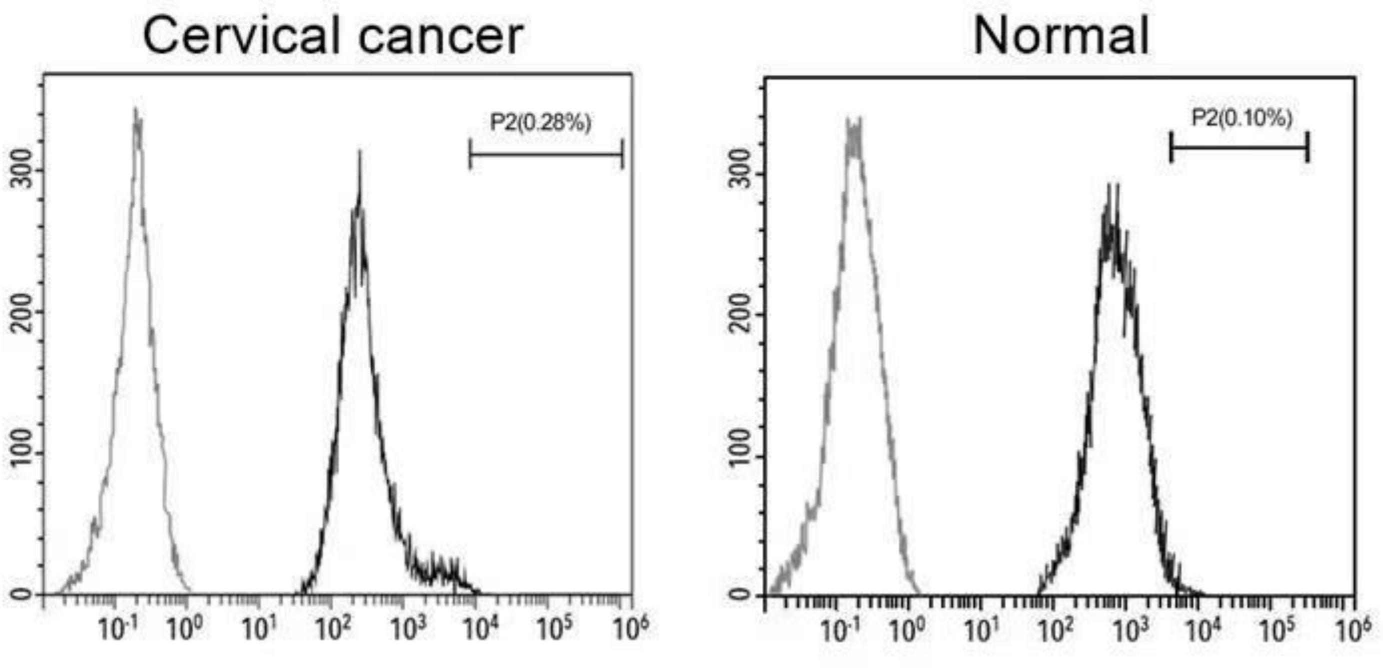

Supplementary Fig. S4. Negligible PD-L1 Surface Expression on NK Cells. Blood-derived NK cells, autologous biopsy-derived malignant squamous cells from HPV+ cervical cancer patients (*n*=24), or normal cervical squamous cells from age-matched healthy HPV-negative female donors (*n*=24) were independently incubated for 24 hours. Then, the NK cells were co-cultured with either the malignant squamous cells or normal cervical squamous cells for 30 min. Representative flow cytometric images displaying negligible PD-L1 expression on NK cells under both co-culture conditions. Isotype control peak depicted in grey. *n*=3 biological replicates × 3 technical replicates.

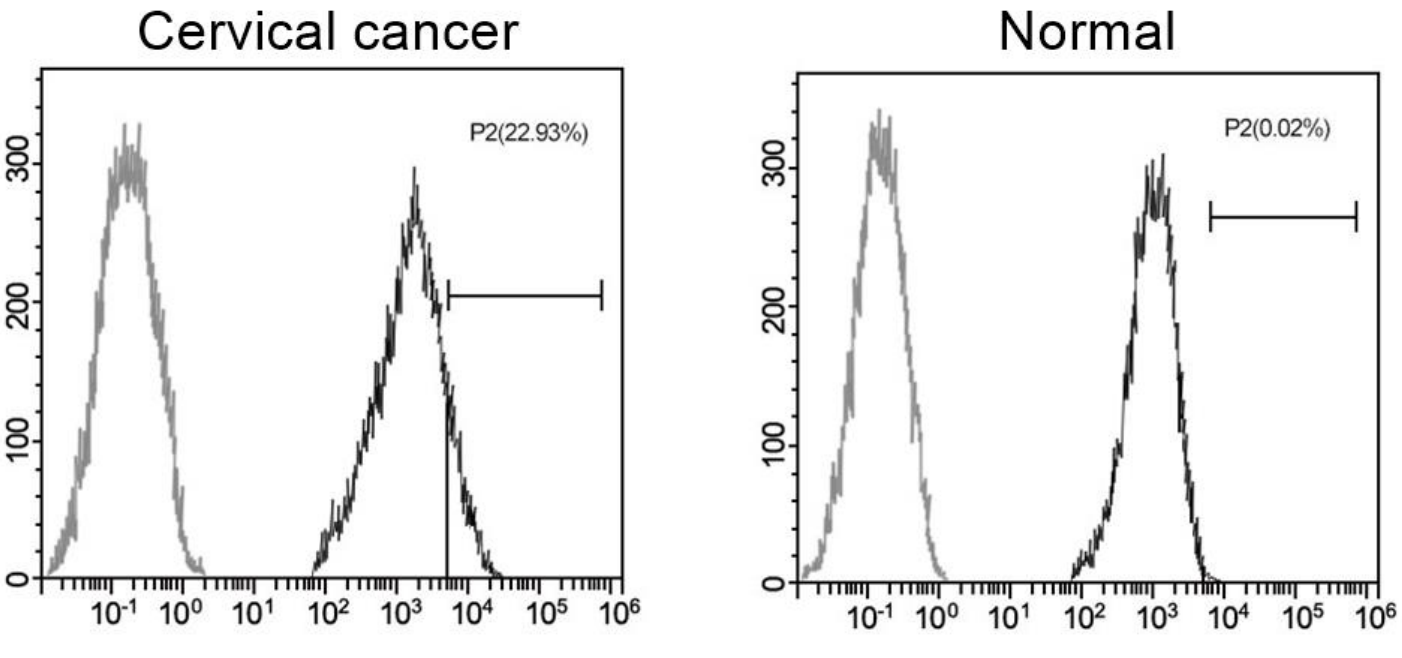

Supplementary Fig. S5. Comparison of PD-L1 Surface Expression on Cervical Cancer Cells and Normal Cells. Representative flow cytometric images displaying positive PD-L1 expression on human tumor-derived cervical cancer cells but negligible PD-L1 expression on normal cervical squamous cells. Isotype control peak depicted in grey. n=3 biological replicates × 3 technical replicates.

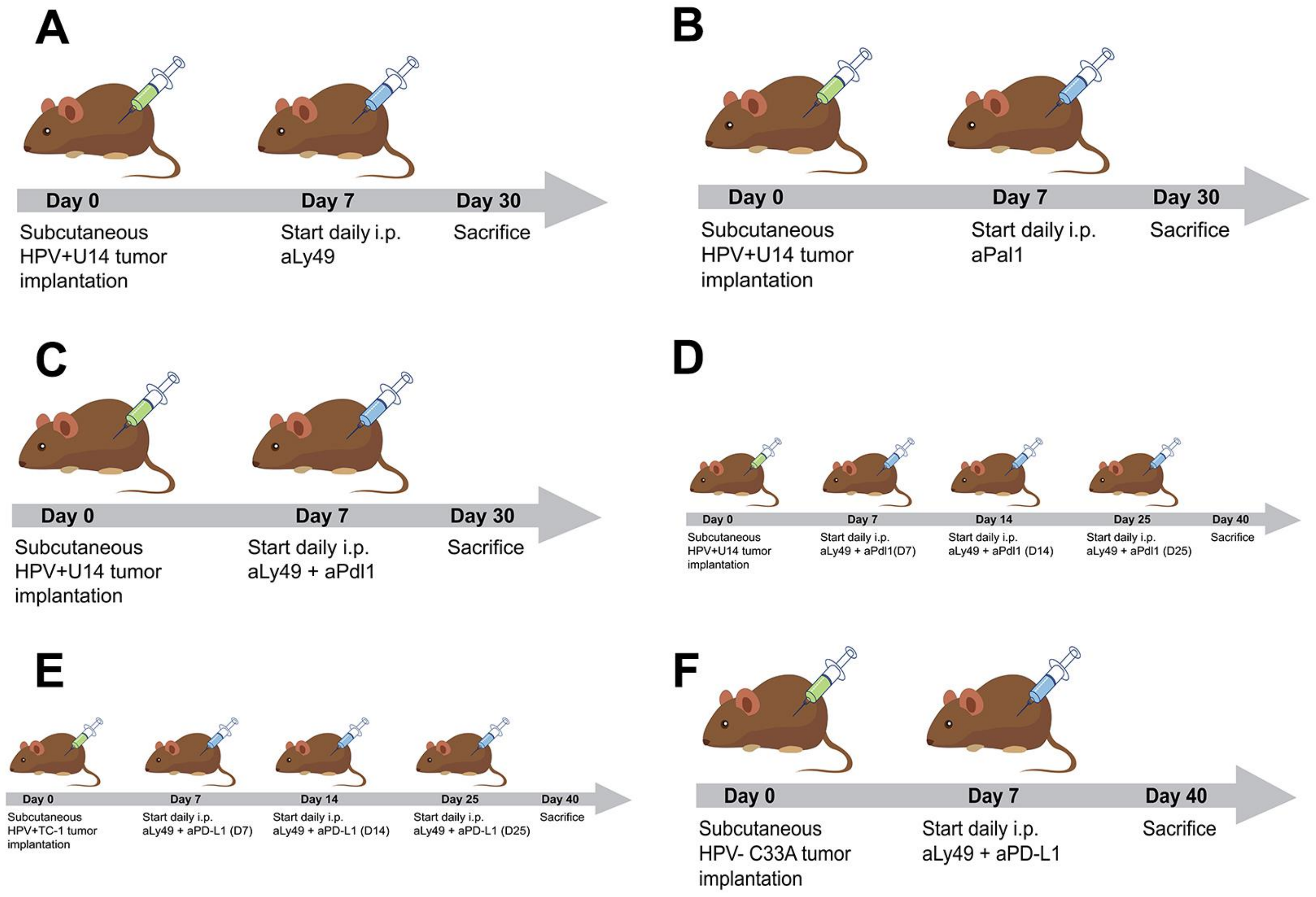

Supplementary Fig. S6. Schematic Diagrams of the Various Mouse Models of Cervical Cancer. Schematic diagrams of the syngeneic murine HPV+ cervical tumor models depicted in (A) Fig. 2A, (B) Fig. 2B, (C) Fig. 2C, (D) Fig. 2D, (E) Fig. 2E, and (F) Supplementary Fig. S8.

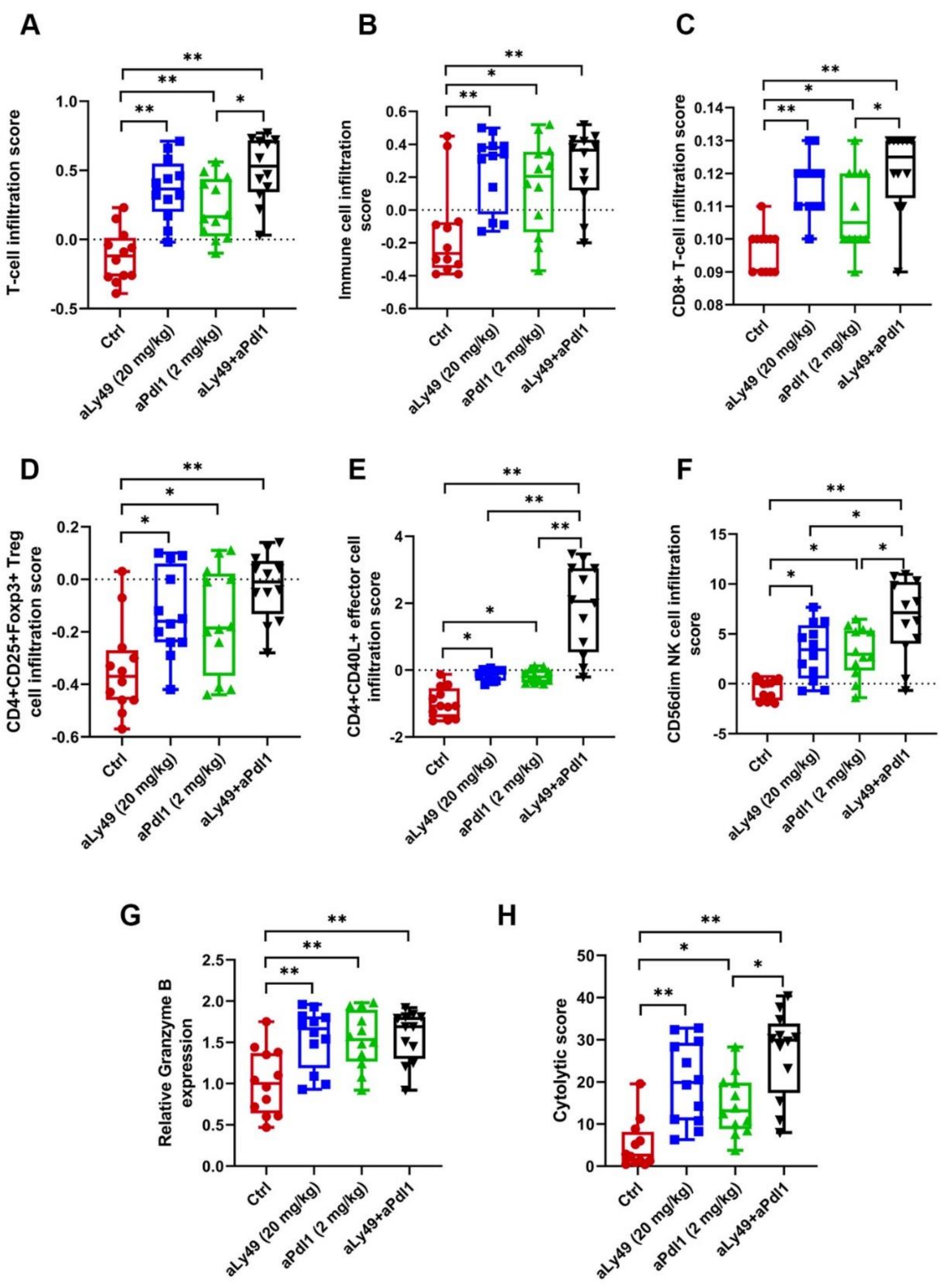

Supplementary Fig. S7. Immune Cell Infiltration Scoring for Harvested Murine HPV+ Cervical Tumors. (A) T-cell infiltration scores (TIS), (B) total immune cell infiltration scores (IIS), (C) CD8+ T cell infiltration scores, (D) CD4+ Treg infiltration scores, (E) CD4+ effector T-cell scores, (F) CD107a+ NK cell scores, (G) relative Granzyme B expression levels, and (H) cytolytic scores among 30 day-old tumors harvested after administration of IgG (control), the avelumab analogue aPdl1 (2 mg/kg daily), or aPdl1 (4 mg/kg daily) intraperitoneally (i.p.) initiated seven days after HPV+ U14 cell implantation. *$P<0.05$, **$P<0.01$ [one-way ANOVA]. All *in vivo* experiments: $n=12$ mice per cohort. All box-and-whisker plots display interquartile ranges (IQRs, boxes) with 1.5 × IQR ranges (whiskers).

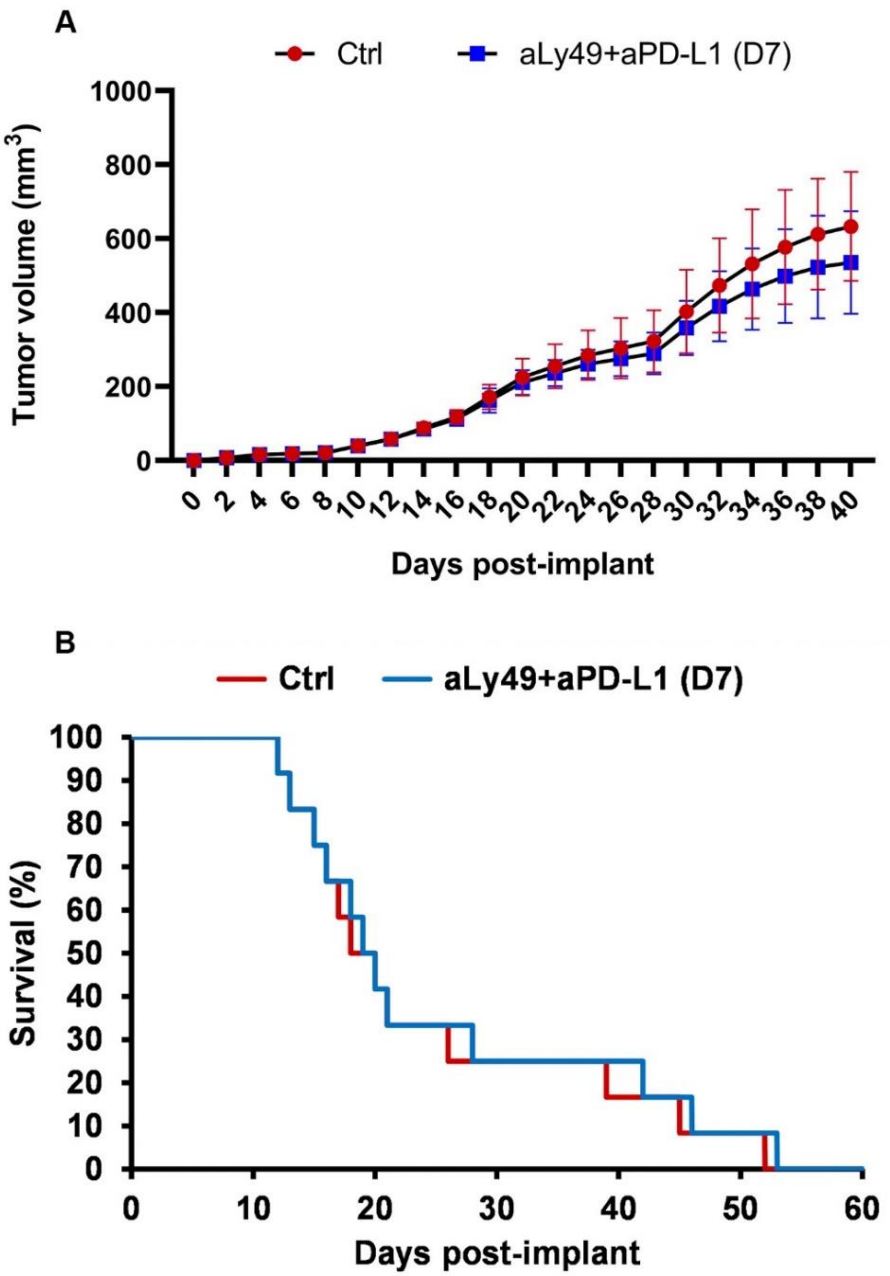

Supplementary Fig. S8. Lirilumab and Avelumab Do Not Display Anti-Tumor Activity in HPV–Mouse Model of Cervical Cancer. Tumor-bearing mice were administered IgG (control), the lirilumab analogue aLy49 (20 mg/kg daily) plus avelumab (aPD-L1, 2 mg/kg daily) intraperitoneally (i.p.) seven days after HPV-negative C33A cervical tumor cell implantation. (A) Tumor volumes and (B) overall survival were analyzed. Tumor volume was calculated as length × width × width/2. *P<0.05, **P<0.01 [(A) one-way ANOVA or (B) log-rank test]. All *in vivo* experiments: n=12 mice per cohort.

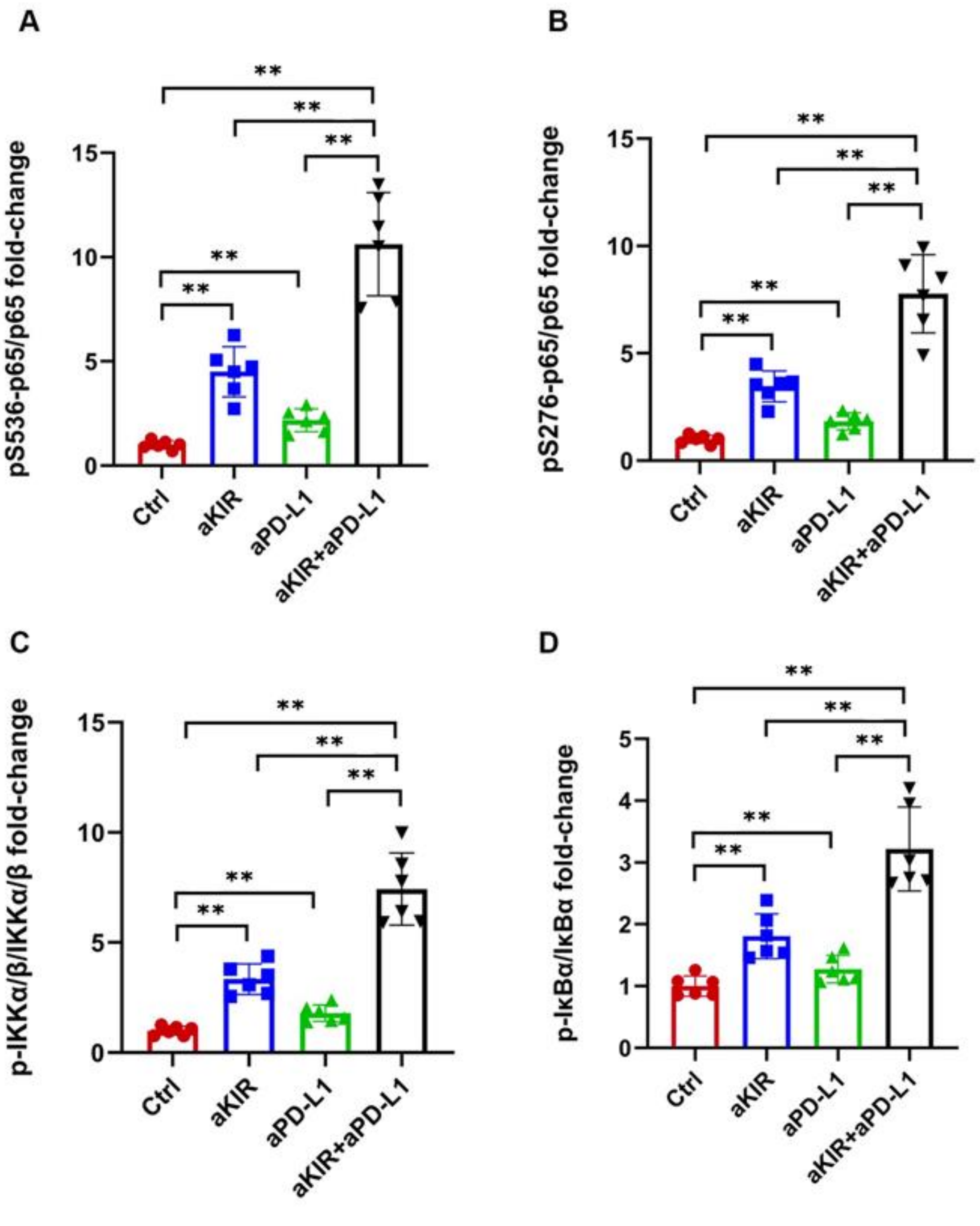

Supplementary Fig. S9. Densitometric Analyses of the Immunoblots Depicted in Fig. 3A. IL-2-primed NK cells were mixed with autologous target cells and stimulated with lirilumab (aKIR) and/or avelumab (aPD-L1) by receptor crosslinking for 4 h. Whole cell lysates from isolated NK cells were immunoblotted for phospho-S536 p65, phospho-S276 p65, total p65, phospho-IKKα/βS176/180, total IKKα/β, phospho-IκBα, total IκBα, and β-actin. Normalized intensities of (A) phospho-S536 p65, (B) phospho-S276 p65, (C) phospho-S176/180 IKKα/β, and (D) phospho-IκBα relative to their total counterparts are reported. **P*<0.05, ***P*<0.01 [one-way ANOVA]. *n*=3 biological replicates×3 technical replicates.

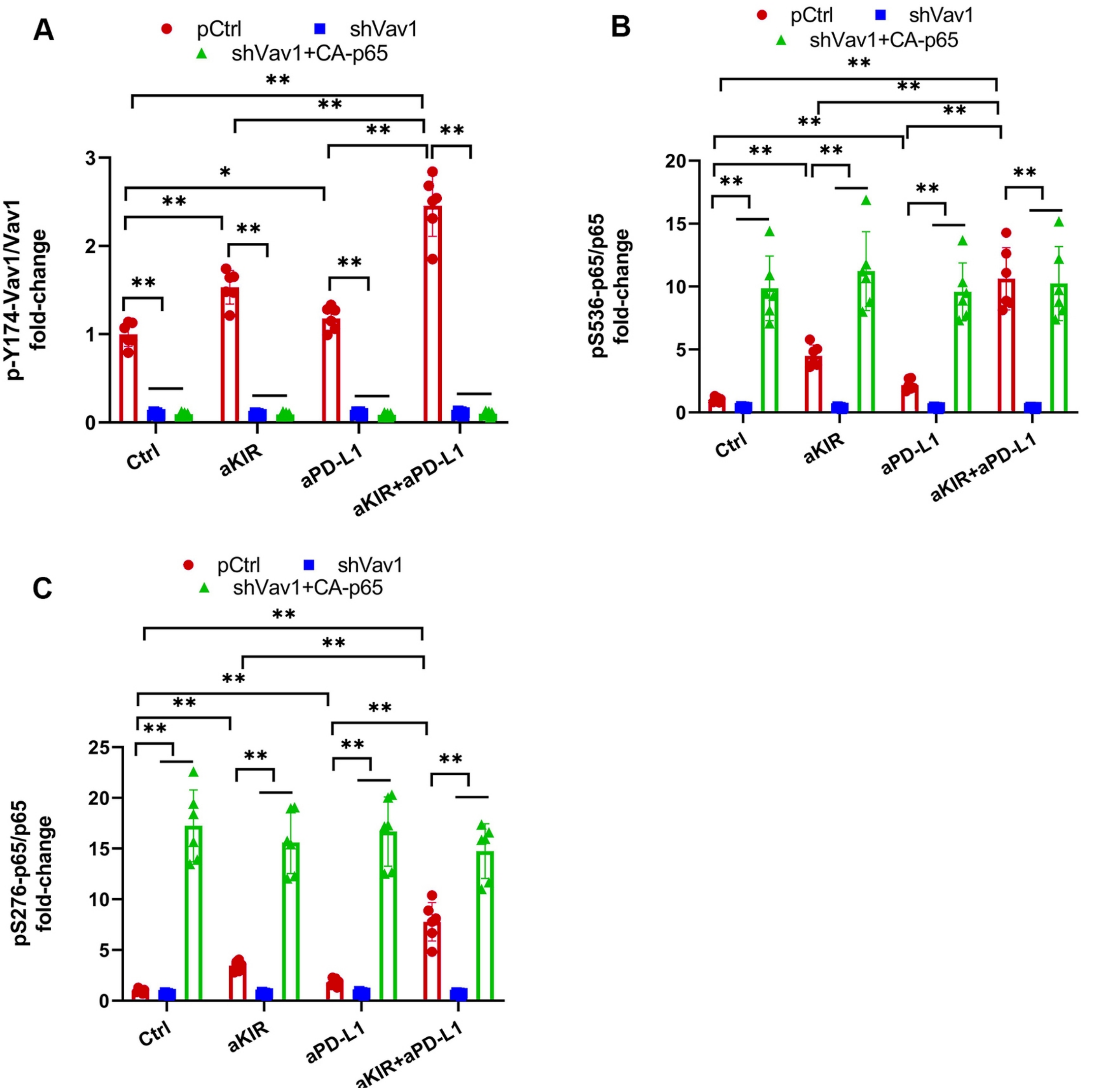

Supplementary Fig. S10. Densitometric Analyses of the Immunoblots Depicted in Fig. 5F. IL-2-primed NK cells transfected with empty plasmid control (pCtrl), anti-Vav1 shRNA (shVav1), and shVav1+constitutively-active p65 phosphomutant p-S536D/S276D p65 (shVav1+CA-p65) were mixed with autologous target cells and stimulated with lirilumab (aKIR) and/or avelumab (aPD-L1) by receptor crosslinking for 4 h. Lysates from isolated NK cells were immunoblotted for phospho-Y174 Vav1, total Vav1, phospho-S536 p65, phospho-S276 p65, total p65, and β-actin. Normalized intensities of (A) phospho-Y174 Vav1, (B) phospho-S536 p65, and (C) phospho-S276 p65 relative to their total counterparts are reported. *P<0.05, **P<0.01 [two-way ANOVA; mAb factor × transfection factor]. n=3 biological replicates×3 technical replicates.
